# Hormonal crosstalk controls cell death induced by kinetin in roots of *Vicia faba* ssp. *minor* seedlings

**DOI:** 10.1038/s41598-023-38641-5

**Published:** 2023-07-19

**Authors:** Andrzej Kaźmierczak, Danuše Tarkowská, Lenka Plačková, Magdalena Doniak, Karel Doležal

**Affiliations:** 1 Department of Cytophysiology, Institute of Experimental Biology, Faculty of Biology and Environmental Protection, University of Łódź, Pomorska 141/143, 90-236 Łódź, Poland; 2 Laboratory of Growth Regulators, Centre of the Region Haná for Biotechnological and Agricultural Research, Institute of Experimental Botany Academy of Sciences of the Czech Republic and Palacký University, Šlechtitelů 27, 78371 Olomouc, Czech Republic; 3 Laboratory of Growth Regulators, Institute of Experimental Botany of the Czech Academy of Sciences, and Faculty of Science Palacký University, Šlechtitelů 27, 78371 Olomouc, Czech Republic; 4 Department of Chemical Biology and Genetics, Center of Region Haná for Biotechnological and Agricultural Research, Faculty of Science, Palacký University, Šlechtitelů 27, 78371 Olomouc, Czech Republic

**Keywords:** Biochemistry, Cell biology, Cell death

## Abstract

Studies of vitality/mortality of cortex cells, as well as of the concentrations of ethylene (ETH), gibberellins (GAs), indolic compounds/auxins (ICs/AUXs) and cytokinins (CKs), were undertaken to explain the hormonal background of kinetin (Kin)-regulated cell death (RCD), which is induced in the cortex of the apical parts of roots of faba bean (*Vicia faba* ssp. *minor*) seedlings. Quantification was carried out with fluorescence microscopy, ETH sensors, spectrophotometry and ultrahigh-performance liquid chromatography tandem mass spectrometry (UHPLC‒MS/MS). The results indicated that Kin was metabolized to the transport form, i.e., kinetin-9-glucoside (Kin9G) and kinetin riboside (KinR). KinR was then converted to *cis*-zeatin (*c*Z) in apical parts of roots with meristems, to *cis*-zeatin riboside (*c*ZR) in apical parts of roots without meristems and finally to *cis*-zeatin riboside 5’-monophosphate (*c*ZR5’MP), which is indicated to be a ligand of cytokinin-dependent receptors inducing CD. The process may be enhanced by an increase in the amount of dihydrozeatin riboside (DHZR) as a byproduct of the pathway of zeatin metabolism. It seems that crosstalk of ETH, ICs/AUXs, GAs and CKs with the *c*ZR5’MP, the *cis*-zeatin-dependent pathway, but not the *trans*-zeatin-dependent pathway, is responsible for Kin-RCD, indicating that the process is very specific and offers a useful model for studies of CD hallmarks in plants.

## Introduction

Cell death (CD), along with the cell division cycle, is the most important process controlling the differentiation of plant^1^ and animal^2^ cells and prokaryotic organisms^3^. According to the recommendation of Galluzzi et al., CD induced by exogenous environmental stimuli (physical and biological)^4^ and by experimentally applied factors such as phytohormones, e.g., 6-benzylaminopurine (BAP)^1^, should be called regulated cell death (RCD), while the process induced by endogenous stimuli should be called programmed cell death (PCD)^2^. These descriptions are consistently applied throughout the paper, while the keywords and most commonly used abbreviations are collected in Table 1.

**Table 1:** List of the keywords and the most frequently used abbreviations.

List	Description
Keywords	auxins, BY-2 cells, cytokinins, ethylene, gibberellins, exogenously regulated cell death, kinetin, ultra-high performance liquid chromatography/mass spectrometry, *Vicia faba* ssp. *minor*
The most frequently used abbreviations	1-aminocyclopropane-1-carboxylic acid (ACC); apical part of roots (APR); abscisic acid (ABA); acridine orange (AO); arabidopsis response regulator (ARR); auxin/auxins (AUX/AUXs); 6-benzylaminopurine (BAP); cytokinin/cytokinins (CK/CKs); cell cycle switch52 A1 (CCS52A1); ethidium bromide (EB); ethylene (ETH); ethylene responsive element binding factors 2 (ERF2); fluorescence intensity (RFI); gibberellic acid (GA_3_); gibberellin/gibberellins (GA/GAs); gibberellin A_20_ (GA_20_); gibberellin A_6_ (GA_6_); gibberellin A_1_ (GA_1_); indole-3-acetic acid (IAA); indole-3-acetic acid inducible 3, short hypocotyl (SHY2/IAA3); indolic compounds (ICs); kinetin (Kin); kinetin externally induced regulated cell death (Kin-ERCD); kinetin riboside (KinR); kinetin-9-glucoside (Kin9G); *ortho*-methoxytopolin-riboside (MeoTR); programmed cell death, (PCD); reactive oxygen species (ROS); regulated cell death (RCD); *cis*-zeatin (*c*Z), *cis*-zeatin riboside (*c*ZR); *cis*-zeatin riboside-*O*-glucoside (*c*ZROG); dihydrozeatin riboside (DHZR); *cis*-zeatin riboside-5´-monophosphate (*c*ZR5’MP)

The induction of CD by kinetin (Kin) was documented in two scientific experimental models, i.e., in the root cortex of faba bean (*Vicia faba* ssp. *minor*) seedlings^5,6^ and in suspension culture of tobacco BY-2 line (*Nicotiana tabacum* L. cv Bright Yellow-2; BY-2) cells^7^. In both systems, authors of studies recommend use of the term regulated CD, i.e., Kin-RCD, for CD induced by Kin.

Kin-RCD in faba bean has been studied for more than 10 years, while the results of Kin-RCD in BY-2 were published recently^7^. The following hallmarks are worth describing:

1. The loss of cellular (plasma, nuclear and endomembrane) membrane integrity manifested by greater leakage of potassium ions and endomembrane potentials, a higher index of fatty acid unsaturation, malformation of the nuclear envelope, lower amounts of total lipids and their lipid peroxides, lower amounts of phospholipids and changes in their composition^5^ were thus far documented in faba bean, while the increase in permeability of membranes to ethidium bromide, which stains nuclei of dying and dead cells from orange to red, respectively, as well as the increase in conductivity of culture solution were observed in both faba bean and BY-2 models^5,7^.
2. Arrest of the cell cycle, chromatin condensation and chromatin fragmentation^8^ were observed in both faba bean and BY-2 models^7,8^. In faba bean, the heterochromatin fraction undergoes condensation, while euchromatin undergoes decondensation^9^ mediated by exo-/endonucleolytic enzymes^6,8,9^. There was also greater activity of the ROS scavenging enzymes^10^ (catalases and superoxide dismutases) as well as reduction in dehydrogenase activities^8^, fluctuation of the activities of H1- and core-histone kinases and serine- and cysteine-dependent proteases and changes in *β*1 proteasome subunit activity, indicating that proteolytic activities played important roles in signal transduction and in the progression of the process.
3. Formation of small and then large acidic lytic vacuoles, reduction of the number of mitochondria^8^, absence of plasmodesmata in the cell walls of living cortex cells surrounding the aerenchyma cavity, as a probable result of larger amounts of callose forming the domains of dying and nondying cells^10^ by clogging plasmodesmata, were thus far documented in faba bean. There were also ROS-dependent morphological malformations of mitochondria^10^, thickening of the walls of nondying cells^10^, formation of micronuclei and/or apoptotic-like (pseudoapoptotic) bodies^6,8^, and elevation of amounts of cellulose and other cell wall-bound sugars^10^. Increases in the amounts of callose and total and cytosolic concentrations of Ca^2+^ ions^7,11^ were also documented*.* However, an increase in the concentration of soluble sugars and a decrease in that of storage sugars in faba bean and BY-2, respectively^7,10^, were observed.
4. An increase in the amount of ACC, a direct precursor of ethylene (ETH)^11^, along with no change in the concentration of protein^6^ and a decrease in the concentration of ATP^9^ have thus far been observed in faba bean. While in BY-2, the loss of microtubule and actin integrity was observed^7^.

Phytohormones occupy a prominent position, playing regulatory roles in plant physiology (although there are few relevant data) and in the induction, transduction and progression of CD^12^. It has been proposed that in animals, cytokinins (CKs) and auxins (AUXs) are novel cancer therapy agents because *ortho*-methoxytopolin-riboside (MeoTR) inhibition of HeLa cell proliferation was synergistically enhanced by indole-3-acetic acid (IAA) without cytotoxic effects^13^. AUXs also act against CD *Arabidopsis thaliana* L. Heynh., by regulating cellulose synthesis^12^.

Another cytokinin, i.e., BAP, induced death of both carrot (*Daucus carota* L.) and *A. thaliana* (L.) Heynh. cells in suspension cultures^1^. In rice (*Oryza sativa*)*,* drought stress triggered the overexpression of senescence-associated genes (*OsSAP*), which have anti-apoptotic-like activity, while abscisic acid (ABA) stimulated the production of anti-apoptotic proteins and reduced the expression of several pro-apoptotic proteins, and jasmonic acid (JA) negatively regulated CD and lesion formation^14^.

Moreover, it was found that salicylic acid (SA) and/or JA and/or ETH mediated resistance against biotrophs and necrotrophs as well as herbivorous insects/animals via CD^15^, while CD induced by hydrogen peroxide increased the amounts of SA and JA, and then CKs^16^, and SA played a crucial role in the reduction of mitochondrial ROS production^12^.

One of the best-known CD processes controlled by phytohormones is related to the initiation of release of the storage material from the aleurone layer of cereals during seed germination. ABA and GAs regulate the activity of secretory tissue. GA-triggering enzymes initiate a program that culminates in CD, while ABA seems to prevent CD. However, GA sensitizes aleurone cells to ROS, a regulator of cell viability^17^.

In dexamethasone-dependent induction of necrotic lesions in transgenic tobacco leaves based on CD, resembling the hypersensitive response, the plant defence against pathogen attack seems to be controlled by CKs, which could act as mediators of plant pathogen interactions^18^.

Aerenhyma formation in flooded sunflour (*Helianthus annuus*) plants is also controlled by hormonally dependent CD, in which ROS manifest a signalling role^12^ and are induced by ETH^19^ and its precursor, i.e., ACC^20^. It seems that ETH, which probably mediates all cases of CD in plants, is the best-characterized PCD hormone^4,18^ and controls CD induced by itself by ethylene responsive element binding factor 2 (ERF2)^12^, as was found in *Petunia* sp.

Based on studies in faba bean, the results of which were published in 2021^21^, using blockers and inhibitors of the cytokinin receptors and enzymes of their metabolism, respectively, such as PI-55, Ad, Ado, CPPU, EGTA, La^3+^, RRed and CS-A, Sorafenib, Syntide-2 and Mek2, inhibitors of RAF kinase as well as RAF-like and MEK2, it was strongly indicated^21^ that the induction of Kin-PCD was related to the conversion of Kin to corresponding ribotides, e.g., 5'-monophosphate ribonucleotides^21,22^. These purine ligands, as indicated earlier^21,22^, seemed to be specific for histidine kinase receptors (AHK2, AHK3 or AHK4) found in the ER membrane of *A. thaliana* (L.) Heynh. and maize (*Zea mays),* which can activate CD^21–23^.

The aim of the present study was to determine the specificity and sites of CD-related activities of hormones. We studied over 40 hormones (Table 2) in one-third (1/3) of apical parts of roots of Ctrl and Kin-treated seedlings during a 96-h experiment. Many of the hormones were not identified (Table 2). Among those identified, we found (i) a Kin-CD-dependent relationship, i.e., showing that Kin-RCD in faba bean was accompanied by changes in hormone amounts compared to Ctrl and (ii) a Kin-CD-independent relationship, where no changes in hormone amounts were observed compared to Ctrl after Kin treatment. The paper presents the results concerning the Kin-CD-dependent hormonal relationship.

**Table 2:** List of cytokinins, gibberellins and auxins analysed in apical parts of roots of *Vicia faba* ssp. *minor* seedlings.

Plant growth hormone	Hormones of cell death-dependent	Hormones below of detection limit < LOD	Hormones of not cell death-dependent
I. Cytokinins			
* Trans-zeatin derivatives*			
*t*Z—*trans* zeatin			** + **
*t*ZOG—*trans* zeatin-*O*-glucoside			** + **
*t*ZR—*trans* zeatin riboside		** + **	
*t*ZROG—*trans* zeatin ríboside-*O*-glucoside		** + **	
*t*Z7G—*trans* zeatin-7-*O*- glucoside		** + **	
*t*Z9G—*trans* zeatin-9-*O*-glucoside		** + **	
*t*ZR5´MP—*trans* zeatin-5'-monophosphate	** + **		
*cis-zeatin derivatives*		** + **	
*c*Z—*cis* zeatin	** + **		
*c*ZOG—*cis* zeatin-*O*-glucoside		** + **	
* c*ZR—*cis* zeatin riboside			** + **
*c*ZROG—*cis* zeatin riboside-*O*-glucoside	** + **		
*c*Z7G—*cis* zeatin-7-*O*-glucoside		** + **	
*c*Z9G—*cis* zeatin-9-*O*-glucoside		** + **	
*c*ZRMP—*cis* zeatin-5'-monophosphate		** + **	
DHZR—dihydrozeatin riboside	** + **		
Isopentenyladenine derivatives			
iP—isopentenyladenine			** + **
iPR—isopentenyladenosine			** + **
iP7G—isopentenyladenosine -7-*O*-glucoside		** + **	
iP9G- isopentenyladenosine -9-*O*-glucoside			** + **
iPRMP—isopentenyladenosine -5'-monophosphate		** + **	
*Benzylaminopurine derivatives*			
BAP—benzylaminopurine		** + **	
BAPR—benzylaminopurine riboside		** + **	
BAP7G—benzylaminopurine-7-*O*-glucoside		** + **	
BAP9G—benzylaminopurine-9-*O*-glucoside		** + **	
BAPRMP—benzylaminopurine riboside 5'-monophosphate		** + **	
*Meta topolin derivatives*			
*m*T—*meta* topolin		** + **	
*m*TR—*meta* topolin		** + **	
*m*T7G—*meta* topolin-7-*O*-glucoside		** + **	
*m*T9G—*meta* topolin-9-*O*-glucoside		** + **	
Para topolins			
*p*T—*para* topolin		** + **	
*p*TR—*para* topolin		** + **	
*p*T7G—*para* topolin-7-*O*-glucoside		** + **	
*p*T9G—*para* topolin-9-*O*-glucoside		** + **	
*Kinetin derivatives*			
Kin—kinetin	** + **		
KR—kinetin riboside	** + **		
K9G—kinetin-9-*O*-glucoside	** + **		
II. Gibberellins			
GA_4_			** + **
GA_5_			** + **
GA_6_	** + **		
GA_7_		** + **	
GA_9_			** + **
GA_15_			** + **
GA_19_			** + **
GA_20_	** + **		
GA_34_		** + **	
GA_51_		** + **	
III. Auxins			
IAA—indole-3-acetic acid	** + **		
oxIAA—2-oxindole-3-acetic acid		** + **	
IAA-Asp—indole-3-acetyl-aspartic acid		** + **	
IAA-Glu-indole-3-acetic acid glucoside		** + **	

To obtain the results of the studies, roots with (i) and without (ii) meristems of untreated (Ctrl) faba bean seedlings and seedlings treated with Kin were used.

The crosstalk between ETH, total indolic compounds (ICs/AUXs) and IAA, total and individual gibberellins (GAs), GA_20_, GA_6_ and GA_1_, as well as several cytokinins (CKs), Kin, KinR, Kin9G, *c*Z, *c*ZR, *c*ZROG, DHZR and *c*ZRMP, was discussed comparing the Ctrl and Kin variants with and without meristems.

## Results

### The concentration of ETH, length of whole roots, weight of one-third of apical parts of roots and numbers of living, dying, and dead cortex cells

**Figure 1:**
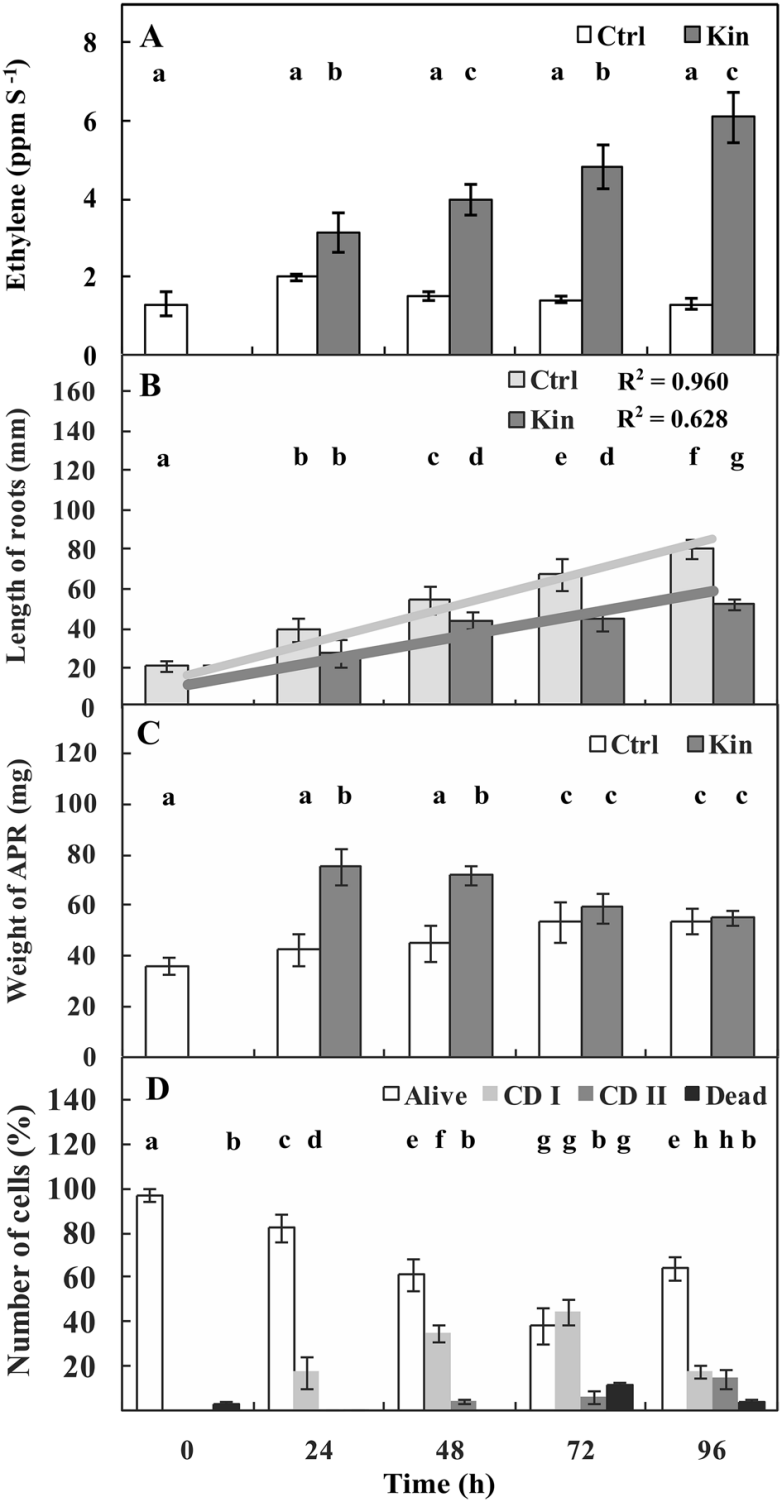
Ethylene concentration (**A**), length of whole roots (**B**) weight of one third (**C**) of apical parts of roots (1/3-APRs) and the number of alive, dying at first (CD I) and second (CD II) stage of cell death (CD) as well as the number of dead cortex cells in (1/3-APRs) (D), the results are in accordance with Kunikowska et al.^8^ of *V. faba* ssp. *minor* seedlings. Bars indicate ± SE of the results from three independent experiments in at least three replicates in which four (number of cell death and ETH concentration; **A**,**D**), at least six (length and weight of roots; **B**,**C**) random samples were analyzed. Each sample consists of at least six seedlings. Means of the results with the same letter above the column are not significantly different at *p* ≤ 0.05.

Measurements indicated that ETH was present in the environment of growing seedlings (Supplementary Fig. S1). The results showed that in the 0-h series, there was approximately 1.5 ppm per seedling, and the ppm values were similar throughout the cultivation time up to 96 h (*p* > 0.05) in control (Ctrl) conditions. Treatment with Kin influenced the ETH level, which was gradually greater day by day and was approximately 6 ppm per seedling at 96 h in the Kin (96-h-Kin) group (Fig. 1A).

The seedling roots at 0 h were approximately 20 mm long, while at 96 h, they were approximately four times longer (*p* ≤ 0.05). During treatment with Kin for 24–48 h, the roots were also gradually and significantly (*p* ≤ 0.05) longer compared to 0 h, but ultimately, i.e., in 96-h-Kin group, they were approximately 3 times longer compared to 0 h and 1/3 shorter compared to 96-h-Ctrl group. This difference was precisely reflected by the R^2^ coefficient, which was 1/3 lower in the Kin series than in the Ctrl series, for which it was 0.960 (Fig. 1B).

The results also indicated that the weight of the apical part of roots (APRs) at 0 h was approximately 35 mg, but in the 96-h-Ctrl group, the roots were approximately 40% heavier (*p* ≤ 0.05). In 24–48-h-Kin, the roots were 2 times heavier (*p* ≤ 0.05) compared to 24–48-h-Ctrl group. However, in the 96-h-Kin group, the roots were approximately 40% (*p* ≤ 0.05) lighter than those in the 24-h-Kin group, and their weight, which was 55 mg per root, was similar (*p* > 0.05) to that in the 96-h-Ctrl group (Fig. 1C).

Treatment of 3-d-old faba bean seedlings with Kin induced the death of cells in the cortex of roots. Analyses using the nuclear fluorescent test showed that in 24–96-h-Ctrl roots, nearly 100% of cells were alive. Treatment with Kin gradually reduced the cell vitality (*p* ≤ 0.05; Fig. 1D). In 72-h-Kin group, there were approximately 45% cells in the first stage of death (CD-I). Moreover, approximately 10% dead cells were also detected. In the 96-h-Kin group, the percentages of live cells showing symptoms of the second stage of death (CD-II) were greater (*p* ≤ 0.05) than that in the 72-h-Kin group by approximately 20 and 10%, respectively (Fig. 1D).

These additional analyses related to ICs/AUXs, AUXs, giberellins (GAs) and cytokinins (CKs) were conducted in 1/3 apical parts of roots, i.e., variants with or without meristems of series of untreated (Ctrl) and treated with Kin faba bean seedlings (Fig. 1).

### The concentration of ICs/AUXs

**Figure 2:**
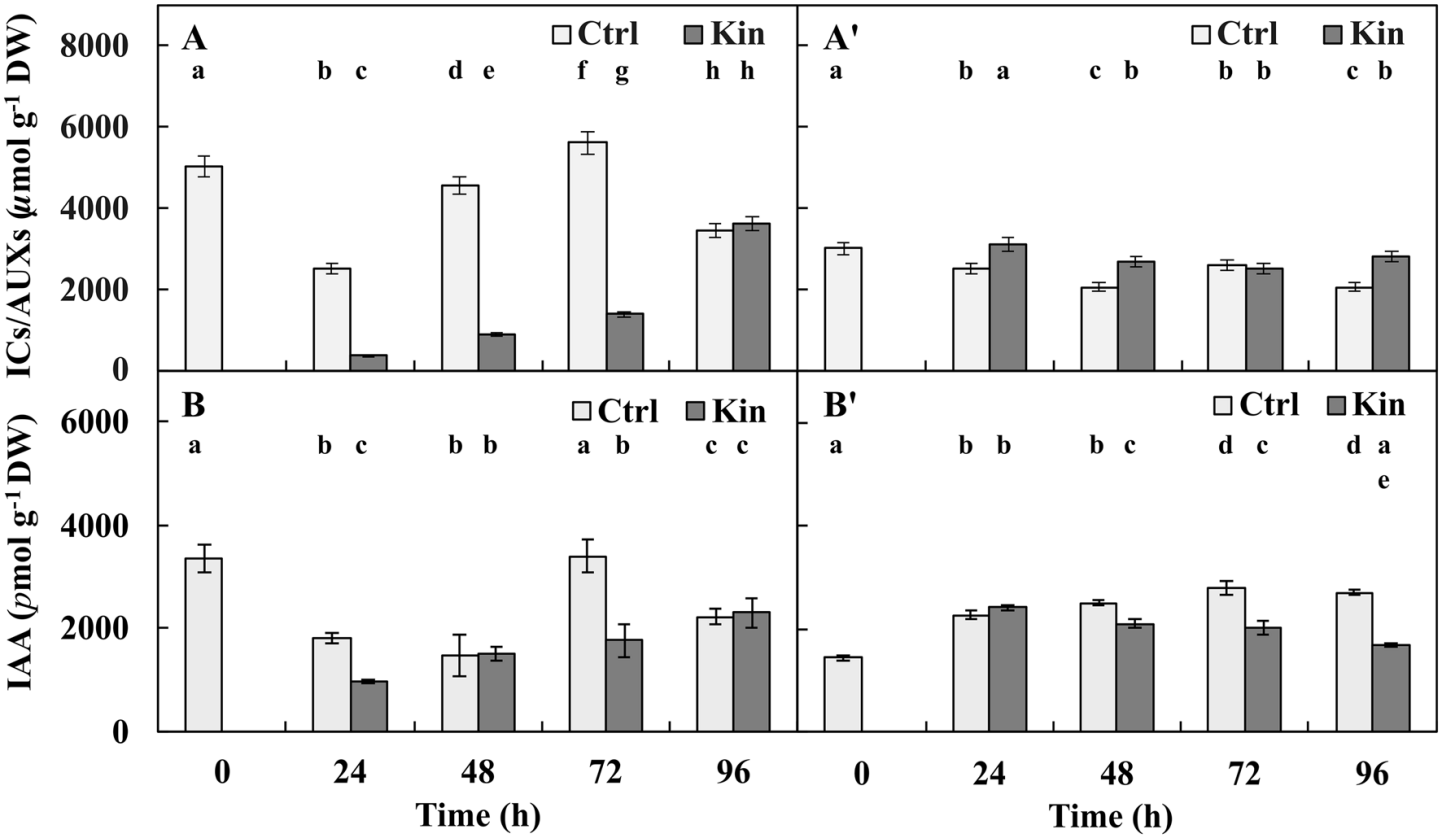
Concentrations of (**A**,**A’**) indolic compounds/auxins (ICs/AUXs) and (**B**,**B’**) indole-3-acetic acid (IAA) in apical parts of roots with (**A**,**B**) and without meristems (**A’**,**B’**) of *V. faba* ssp. *minor* seedlings treated with Kin. Bars indicate ± SE of the results from three (AUXs) or two (IAA) biological replicates, in which three random samples were analyzed. Each sample consists of at least six seedlings. Means of the results with the same letter under the column are not significantly different at *p* ≤ 0.05.

In the variant with meristems in the 24–96-h-Ctrl series, the μmol g^−1^ DW of ICs/AUXs fluctuated (*p* ≤ 0.05) and was observed to be 2.25 times less (*p* ≤ 0.05) in the 24-h than in the 72-h group. In the 24–96-h-Kin series of this variant, there were greater (*p* ≤ 0.05) values every day, and there were 10 times greater (*p* ≤ 0.05) values at 96 h compared to 24 h. On average, the concentrations of ICs/AUXs in 24–96-h-Ctrl and Kin series were 1.25 and 3.25 times lower (*p* ≤ 0.05), respectively, compared to 0 h (Fig. 2A).

In the variant without meristems, the μmol g^−1^ DW of ICs/AUXs fluctuated (*p* ≤ 0.05), and in the Ctrl series, the lowest values were observed at 48 and 96 h, with the greatest values observed at 24 and 72 h, while in the Kin series, the lowest value occurred for 72-h-Ctrl group, with the greatest value found for 24-h-Kin. On average, the concentrations of ICs/AUXs in 24–96-h-Ctrl and Kin series in this variant were approximately 1.25 and 1.1 times lower (*p* ≤ 0.05), respectively, compared to 0 h (Fig. 2A’).

The average concentration of ICs/AUXs in the variant without meristems in the 24–96-h-Kin series was approximately 1.4 times greater (*p* ≤ 0.05) than that in the 24–96-h-Ctrl series of this variant, while compared to the 24–96-h-Ctrl and 24–96-h-Kin series of the variant with meristems, the values were approximately 3 and 1.5 times lower (*p* ≤ 0.05), respectively (Fig. 2A,A’).

### The concentration of IAA

The profile of IAA concentration in the variant with meristems was similar to that observed for ICs/AUXs. In the 24–96-h-Ctrl series, the concentration of IAA fluctuated (*p* ≤ 0.05) and was the lowest at 48 h and 72 h (Fig. 2A). In the 24–96-h-Kin series of this variant, there was greater (*p* ≤ 0.05) IAA every day. In the 96-h-Kin group, the IAA concentration was 2 times greater (*p* ≤ 0.05) than that in the 24-h-Kin group. On average, in the 24–96-h-Ctrl and Kin series, IAA was approximately 1.5 and 2.0 times lower (*p* ≤ 0.05), respectively, than that at 0 h (Fig. 2B).

In the variant without meristems in the 24–96-h-Ctrl and Kin series, there was approximately 1.2 times greater (*p* ≤ 0.05) and approximately 1.4 times lower IAA every day compared to 24 h, respectively. On average, the concentrations in 24–96-h-Ctrl and Kin were approximately 1.75 and 1.5 times greater (*p* ≤ 0.05), respectively, compared to 0 h (Fig. 2B’).

In the variant with meristems in the 24–96-h-Kin series, the average concentration of IAA was approximately 1.25 times lower (*p* ≤ 0.05) compared to the 24–96-h-Ctrl of this variant and was also approximately 1.25 times greater (*p* ≤ 0.05) and similar (*p* > 0.05), respectively, compared to the 24–96-h-Ctrl and 24–96-h-Kin series of the variant without meristems (Fig. 2B, B’).

### The concentration of GAs

**Figure 3:**
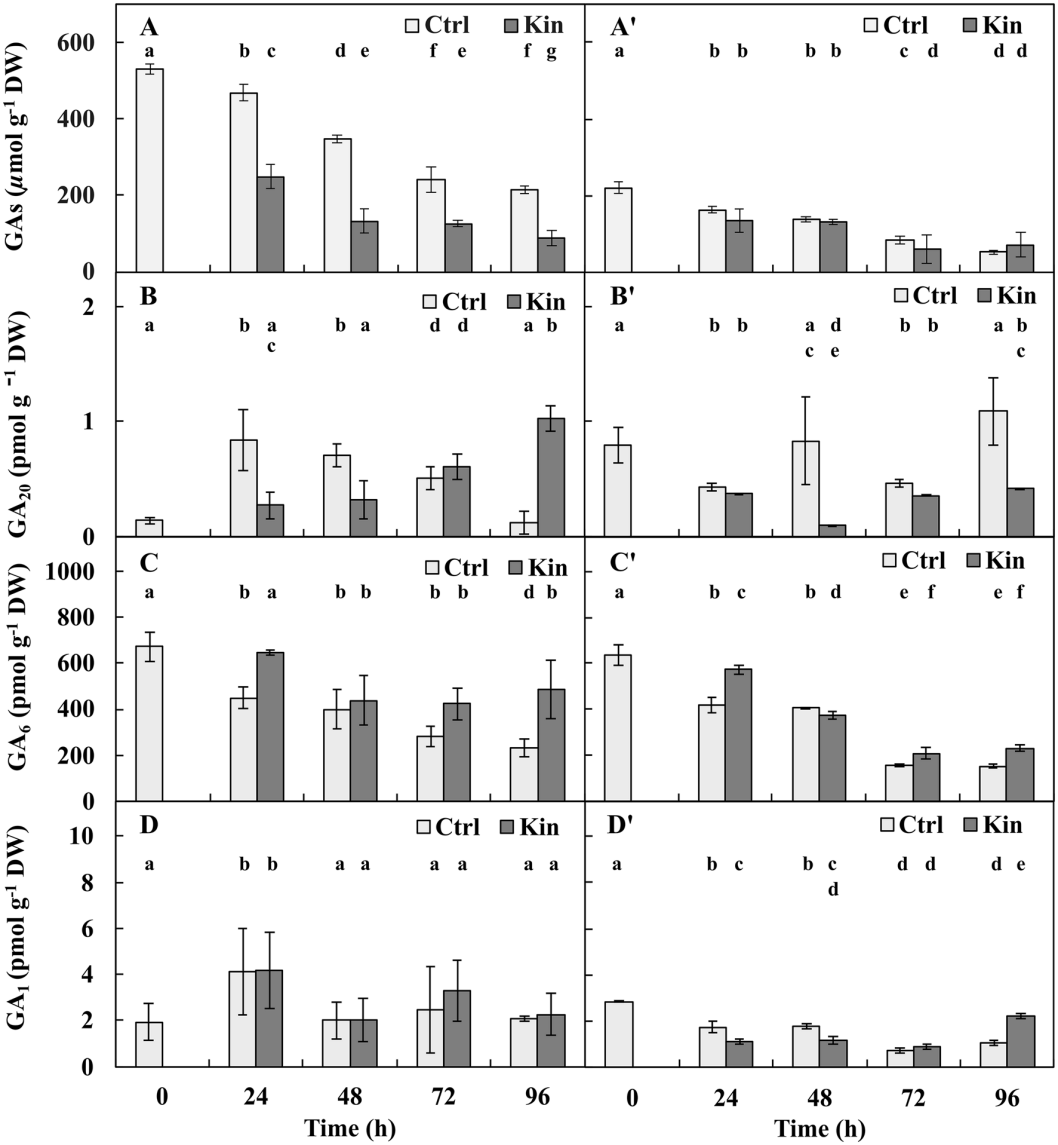
Concentrations of (**A**,**A’**) gibberellins (GAs) (**B**,**B’**) gibberellin A20 (GA_20_), (**C**,**C’**) gibberellin A6 (GA_6_) and (**D**,**D’**) gibberellin A1 (GA_1_) in apical parts of roots with (**A**–**D**) or without (**A’**–**D’**) meristems of *V. faba* ssp. *minor* seedlings treated with Kin. Bars indicate ± SE of the results from the from three biological replicates, in which three random samples for total GAs and two for other GAs were analysed. Each sample consists of at least six fragments of roots. Means of the results with the same letter under the column are not significantly different at *p* ≤ 0.05.

*The concentration of total GAs* in the variant with meristems in 24–96-h-Ctrl and Kin series was gradually lowered (*p* ≤ 0.05), and at 96 h there were approximately 2.25 and 3.0 times less GAs, respectively, compared to 24 h. The average concentrations in the 24–96-h-Ctrl and 24–96-h-Kin series were lower (*p* ≤ 0.05) by approximately 1.75 and 3.5 times, respectively, compared to 0 h (Fig. 3A).

In the variant without meristems (Fig. 3A’), the same profile of the GA concentrations was observed as in the above variant (Fig. 3A). In the 24–96-h-Ctrl and Kin series, the concentrations of total GAs gradually decreased (*p* ≤ 0.05), and at 96 h, they were approximately 3.25 and 2 times lower (*p* ≤ 0.05) than those in the 24-h group. The average concentration of GAs in the 24–96-h-Ctrl and Kin series was approximately 2 times lower (*p* ≤ 0.05) than that at 0 h (Fig. 3A’).

The average concentration of GAs in the 24–96-h-Kin series in the variant without meristems was similar (*p* > 0.05) to the 24–96-h-Ctrl series in the variant of roots with meristems, and it was approximately 3 and 1.5 times lower, respectively (*p* ≤ 0.05), compared to 24–96-h-Ctrl and Kin series of the variant with meristems (Fig. 3A,A’).

*The concentration of GA*_*20*_, a biosynthetic precursor of GAs belonging to the 13-hydroxylation pathway, in the variant with meristems in the 24–96-h-Ctrl series gradually decreased, and at 96 h, it was approximately 7 times lower (*p* ≤ 0.05) than at 24 h. In the respective 24–96-h-Kin series, its concentration was gradually increased and was approximately 2.5 times greater (*p* ≤ 0.05) at 96 h than at 24 h. On average, the concentration of GA_20_ in the 24–96-h-Ctrl and Kin series in the variant with meristems was approximately 4 times greater than that at 0 h (Fig. 3B).

In the variant without meristems in the 24–96-h-Ctrl series, the concentration of GA_20_ fluctuated, and it was the lowest at 24 h (*p* ≤ 0.05) compared to 96 h. In the respective 24–96-h-Kin series, its concentration also fluctuated, and it was the lowest at 48 h, which was 4 times lower (*p* ≤ 0.05) than that at 96 h. On average, the concentration of GA_20_ in 24–96-h-Ctrl was similar (*p* > 0.05), while in 24–96-h-Kin, it was approximately 2 times lower (*p* ≤ 0.05) compared to the 0 h timepoint of this variant (Fig. 3B’).

The average concentration of GA_20_ in the 24–96-h-Kin series was approximately 2 times lower (*p* ≤ 0.05) than that in the 24–96-h-Ctrl series of the variant without meristems and approximately 1.75 times lower (*p* ≤ 0.05) than that in the 24–96-h-Ctrl and Kin series of the variant with meristems (Fig. 3B,B’).

*The concentration of GA*_*6*_, one of the physiologically active gibberellins, in the 24–96-h-Ctrl series of the variant with meristems lowered day by day, and it was lower (*p* ≤ 0.05) by approximately 2 times at 24 h compared to 96 h. In the respective Kin series, the concentration of GA_6_ fluctuated and was the lowest at 48 and 72 h, and it was approximately 1.5 times lower (*p* ≤ 0.05) compared to the greatest concentration at 24 h for that variant. On average, the concentrations of GA_6_ in the 24–96-h-Ctrl series and in the 24–96-h-Kin series were approximately 2 and 1.25 times lower (*p* ≤ 0.05), respectively, than that at 0 h (Fig. 3C).

In the variant without meristems, the concentrations of GA_6_ in the 24–96-h-Ctrl and Kin series were also lower day by day, and at 24 h, the concentrations were approximately 2.5 and 2.75 times lower than at 96 h. On average, the concentration of GA_6_ in the 24–96-h-Ctrl series was approximately 2.25 and 1.77 times lower (*p* ≤ 0.05), respectively (Fig. 3C’).

The average concentration of GA_6_ in the 24–96-h-Kin series of the variant without meristems was 1.25 (*p* ≤ 0.05) greater than that in the 24–96-h-Ctrl of this variant, yet similar (*p* > 0.05) and approximately 1.5 times lower (*p* ≤ 0.05) than those in the 24–96-h-Ctrl and Kin series of the variant with meristems, respectively (Fig. 3C,C’).

*The concentration of GA*_*1*_, a direct and physiologically active product of GA_20_ metabolism, in the variant with meristems in the 24–96-h-Ctrl and Kin series fluctuated (*p* ≤ 0.05) according to a similar scheme. The lowest value at 48 h was 2 times lower (*p* ≤ 0.05) than the greatest value at 24 h. On average, the concentrations of GA_1_ in the 24–96-h-Ctrl and Kin series were approximately 1.25 and 1.5 times greater (*p* ≤ 0.05), respectively, than that in the 0-h group (Fig. 3D).

In the variant without meristems, the concentrations of GA_1_ in the 24–96-h-Ctrl and Kin series also fluctuated (*p* ≤ 0.05), and the lowest values at 72 h were 2 times lower (*p* ≤ 0.05) than the greatest values observed for 24-h-Ctrl and 96-h-Kin groups. On average, the concentration of GA_1_ in 24–96-h-Ctrl and Kin series was similar, and it was approximately 2 times lower (*p* ≤ 0.05) compared to 0 h (Fig. 3D).

The average concentration of GA_1_ in the 24–96-h Kin series of the variant without meristems was similar (*p* > 0.05) to that in the 24–96-h-Ctrl of this variant and approximately 2 times lower (*p* ≤ 0.05) than those in the 24–96-h-Ctrl and Kin series of the variant with meristems (Fig. 3D, D’).

### The amounts of CKs

**Figure 4:**
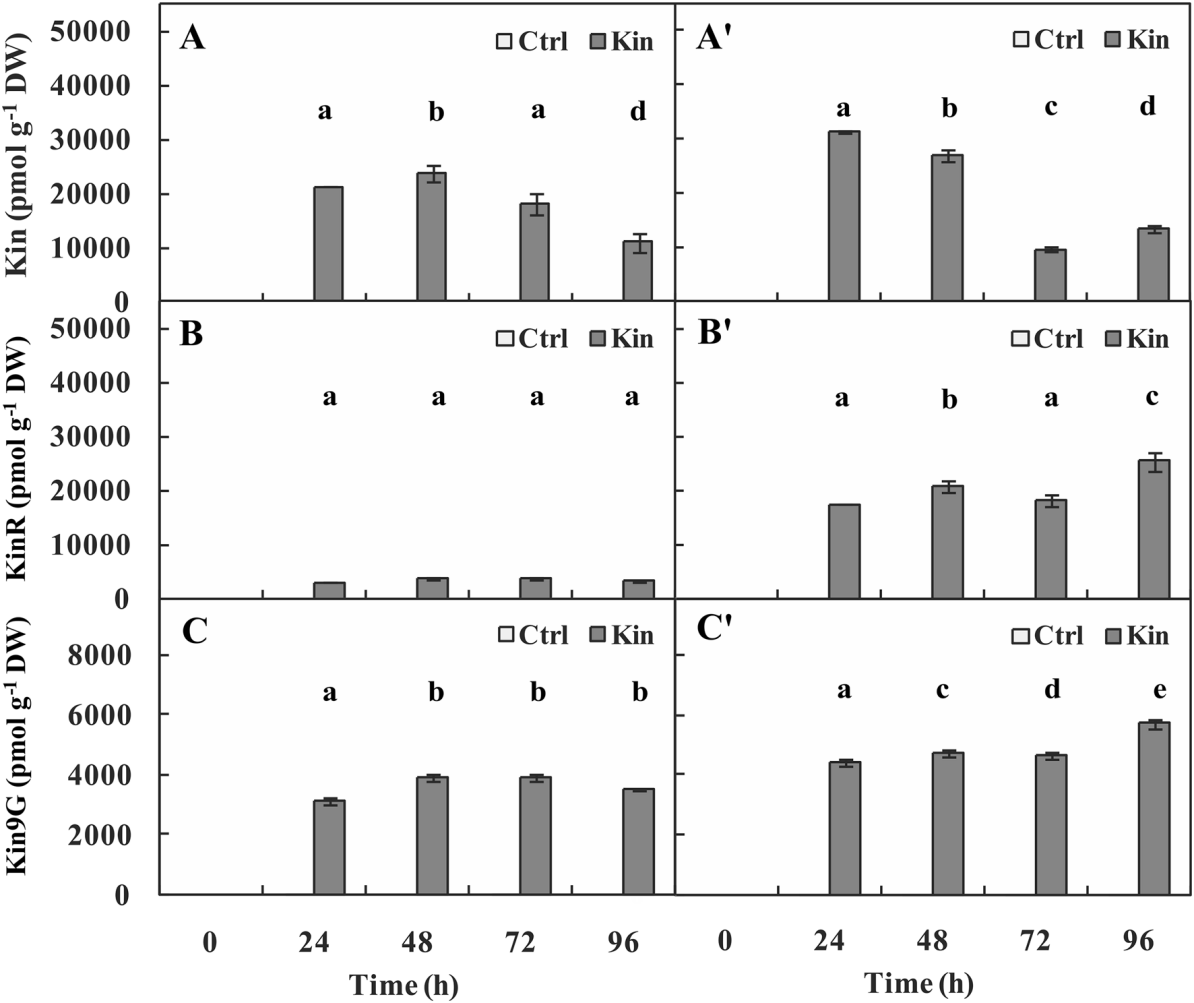
Concentration of (**A**,**A’**) kinetin (Kin) and its derivatives: (**B**,**B’**) kinetin riboside (KinR) and (**C**,**C’**) kinetin-9-*O*-glucoside (Kin9G) in apical parts of roots with (**A**–**C**) or without (**A’**–**C’**) meristems of *V. faba* ssp. *minor* seedlings treated with Kin. Bars indicate ± SE of the results from three independent experiments in which three random samples were analyzed. Each sample consists of at least six fragments of roots. Means of the results with the same letter above the column are not significantly different at *p* ≤ 0.05.

*The concentration of Kin* in apical parts of roots with meristems in the 24–96-h-Ctrl series was detected only at 24, 48 and 72 h. The lowest concentration was observed at 48 h and was approximately 7 times lower (*p* ≤ 0.05) than that at 24 h. In the respective Kin series, the concentration of Kin gradually decreased (*p* ≤ 0.05), and was approximately 2 times (*p* ≤ 0.05) lower at 96 h than at 24 h (Fig. 4A).

In the variant without meristems, Kin was not detected in 0–96-h-Ctrl, while in the respective Kin series, the concentration of Kin decreased daily until 96 h, when it was lower (*p* ≤ 0.05) by approximately 2.25 times compared to 24 h (Fig. 4A’).

The average concentration of Kin in 24–96-h-Kin in the variant without meristems was approximately 600 times greater (*p* ≤ 0.05) and similar (*p* > 0.05) compared to the 24–96-h-Ctrl and Kin series of the variant with meristems, respectively (Fig. 4A,A’).

*The KinR* in apical parts of roots with meristems in the 0–96-h-Ctrl series was detected only at 24 h and 72 h. In the respective Kin series, the concentration of Kin fluctuated. The lowest value was observed at 24 h, which was 1.25 times lower (*p* ≤ 0.05) than that at 72 h (Fig. 4B).

In the variant without meristems, the concentration of KinR in the 24–96-h-Ctrl series also fluctuated between the lowest values at 24 h and the greatest values at 48 h. In the 24–96-h Kin series, the concentration of KinR gradually escalated, and at 96 h, it was approximately 1.25 times greater (*p* ≤ 0.05) than that at 24 h (Fig. 4B’).

The average concentration of KinR was the greatest among the studied hormones, and in the 24–96-h-Kin series of the variant without meristems, it was 1,750 times greater (*p* ≤ 0.05) compared to the 24–96-h-Ctrl series of this variant, approximately 12,500 times greater (*p* ≤ 0.05) compared to 24–96-h-Ctrl and approximately 5.75 times greater (*p* ≤ 0.05) compared to the 24–96-h-Kin series of the variant with meristems (Fig. 4B,B’).

*Kin9G* in apical parts of roots with meristems in the 0–96-h-Ctrl series was detected only at 24 h. In the respective Kin series, the concentration of Kin9R was gradually enhanced and was approximately 1.25 times greater at 96 h than at 24 h (Fig. 4C).

In the variant without meristems, the concentration of Kin9R in the 24–96-h-Ctrl series also fluctuated (*p* ≤ 0.05) between the lowest value at 96 h and the greatest value at 24 h. At 0 h, this factor was not detected. In 24–96-h Kin, the concentration of Kin9R was gradually greater, and it was approximately 1.25 times greater at 96 h than at 24 h (Fig. 4C’).

The average concentration of Kin9R in the 24–96-h Kin series of the variant without meristems was approximately 1,250 times greater (*p* ≤ 0.05) than that in the 24–96-h-Ctrl series of this variant, approximately 17,500 times greater (*p* ≤ 0.05) than that in the 24–96-h-Ctrl and approximately 2 times greater than that in the 24–96-h Kin series of the variant with meristems (Fig. 4C,C’).

**Figure 5:**
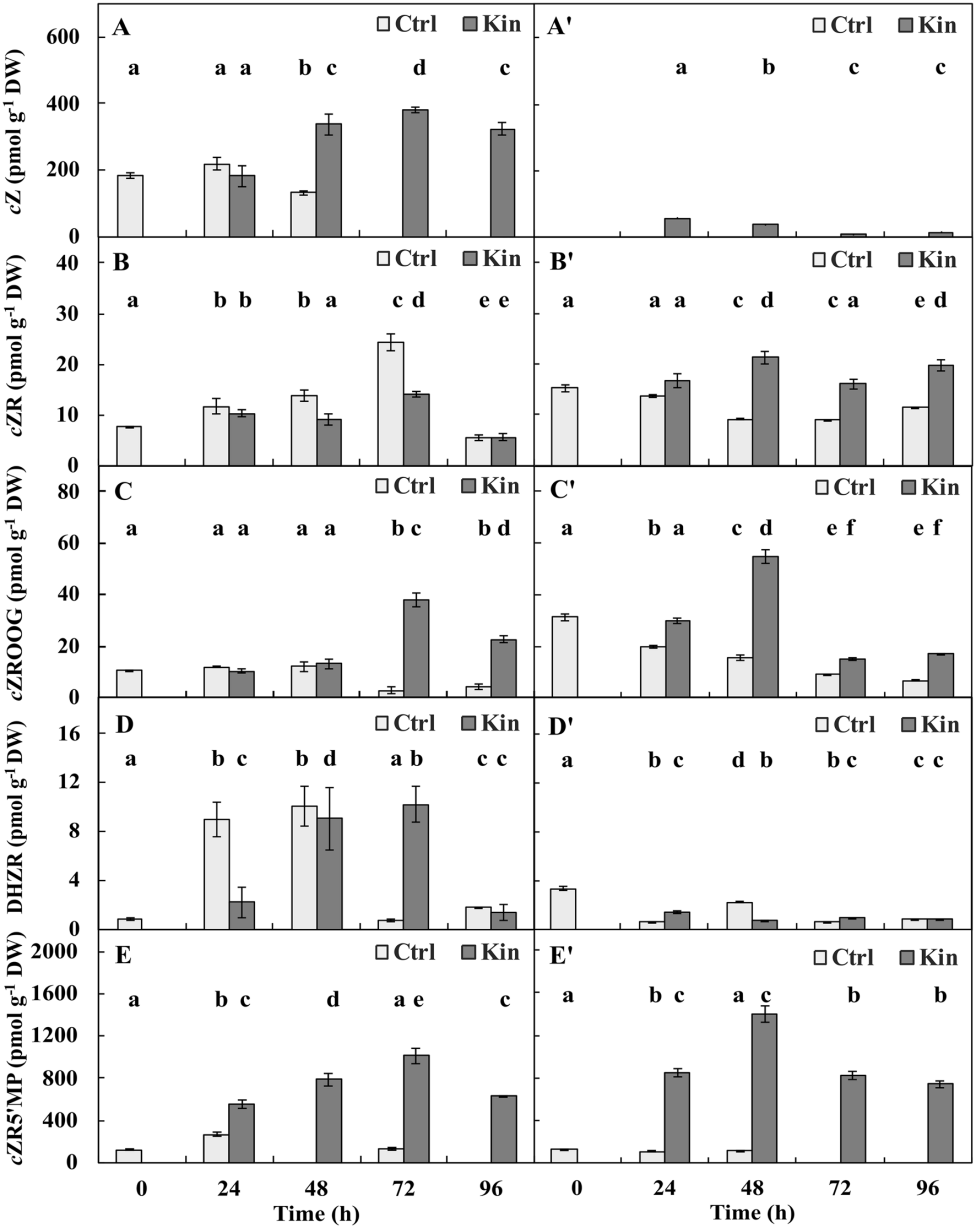
Concentrations of (**A**,**A’**) *cis*-zeatin (cZ) and its derivatives: (**B**,**B’**) *cis*-zeatin riboside (*c*ZR), (**C**,**C’**) *cis*-zeatin riboside-*O*-glucoside (cZROG), (**D**,**D’**) dihydrozeatin riboside (DHZR) and (**E**,**E’**) *cis*-zeatin-5'-monophosphate (*c*ZR5’MP) in apical parts of roots with (**A**–**E**) or without (**A**–**D**) meristems of *V. faba* ssp. *minor* seedlings treated with Kin. Bars indicate ± SE of the results from three biological replicates, in which three random samples were analysed. Bars indicate ± SE of results from three independent experiments repeated three times each. Means of the results with the same letter above the column are not significantly different at *p* ≤ 0.05.

*The cZ* in the variant with meristems was detected at 0-h and 24–48-h-Ctrl group. In the 24-h-Ctrl group of this variant, it was approximately 1.75 greater (*p* ≤ 0.05) than that in the 48-h-Ctrl group. In the respective Kin series, the concentration of *c*Z was gradually greater and was approximately 1.75 times greater at 96 h than at 24 h. On average, the concentrations of *c*Z in the 24–96-h-Ctrl and 24–96-h-Kin series were similar (*p* > 0.05) and 1.75 times greater (*p* ≤ 0.05), respectively, than that in the 0-h group (Fig. 5A).

In the variant without meristems in the 0-h group and 24–96-h-Ctrl series, *c*Z was not identified. In the respective 24–96-h-Kin series, the concentration of *c*Z was gradually lower, and in 24-h-Kin group, it was approximately 7.0 and 3.5 times greater (*p* ≤ 0.05), respectively, compared to 72- and 96-h-Kin group (Fig. 5A’).

The average amount of *c*Z in 24–96-h-Kin series in the variant without meristems was approximately 5 and 10 times lower (*p* ≤ 0.05) than that in the 24–96-h-Ctrl and 24–96-h-Kin series in the variant with meristems, respectively (Fig. 5A,A’).

*The concentration of cZR* in the variant with meristems in the 24–96-h-Ctrl and 24–96-h-Kin series fluctuated, with the lowest value at 96 h, which was 5 and 3 times lower (*p* ≤ 0.05) than the greatest values at 72-h-Ctrl and Kin groups, respectively (Fig. 5B). On average, the concentration of *c*ZR in 24–96-h-Ctrl and in 24–96-h-Kin series was approximately 1.75 (*p* ≤ 0.05) and 1.25 times greater (*p* ≤ 0.05), respectively, compared to 0 h (Fig. 5B).

In the variant without meristems in the 24–96-h-Ctrl and 24–96-h-Kin series, the concentration of *c*ZR fluctuated. In the 24–96-h-Ctrl series, the lowest (*p* ≤ 0.05) expression was observed at 48 and 72 h, and it was 1.5 times lower (*p* ≤ 0.05) than that at 24 h. On average, the concentration of *c*ZR in the 24–96-h-Ctrl series was approximately 1.5 lower (*p* ≤ 0.05) and 1.25 times greater (*p* ≤ 0.05) than that in the 24–96-h-Kin series at 0 h (Fig. 5B’).

The average concentration of *c*ZR in the 24–96-h-Kin series in the variant without meristems was approximately 1.75 times greater (*p* ≤ 0.05) than that in the 24–96-h-Ctrl series of this variant and approximately 1.25 and 2 times greater (*p* ≤ 0.05) than in the 24–96-h-Ctrl and 24–96-h-Kin series, respectively (Fig. 5B,B’).

*The concentration of cZROG* in the variant with meristems in the 24–96-h-Ctrl series gradually decreased, and it was 3 and 4 times lower at 72 and 96 h, respectively (*p* ≤ 0.05), compared to 24 and 48 h (Fig. 5C). In the respective Kin series, the concentration of *c*ZROG was gradually enhanced and was approximately 3.5 and 2 times greater at 72 h and 96 h, respectively, than at 24 h. On average, the concentration of *c*ZROG in the 24–96-h-Ctrl and Kin series was approximately 1.5 times lower (*p* ≤ 0.05) and 2 times greater (*p* ≤ 0.05), respectively, than that in the 0-h group (Fig. 5C).

In the variant without meristems in the 24–96-h-Ctrl series, the concentration of *c*ZROG gradually decreased, and at 96 h, when there was approximately 7.5 pmol g^−1^ DW, it was 4.5 times lower (*p* ≤ 0.05) than that in the 24-h-Ctrl group (Fig. 5C). In the respective Kin series, the concentration of *c*ZROG fluctuated and was lowest at 72 h, when there was approximately 15 pmol g^−1^ DW, and it was 3.5 times lower than that at 48 h, when the concentration of *c*ZROG was the greatest. On average, the concentration of *c*ZROG in 24–96-h-Ctrl and in 24–96-h-Kin was approximately 12.75 pmol g^−1^ DW, and it was approximately 2.5 times lower (*p* ≤ 0.05) and similar (*p* > 0.05) compared to 0 h, when there was approximately 30 pmol g^−1^ DW (Fig. 5C’).

On average, the concentration of *c*ZROG in the 24–96-h-Kin series in the variant without meristems was approximately 30 pmol g^−1^ DW, which was 2.25 times greater (*p* ≤ 0.05) than that in the 24–96-h-Ctrl series in this variant and approximately 3.75 pmol g^−1^ DW, 1.5 times greater (*p* ≤ 0.05) than that in the 24–96-h-Ctrl and 24–96-h-Kin series in the variant with meristems, respectively (Fig. 5C,C’).

*The concentration of DHZR* in the variant with meristems in the 24–96-h-Ctrl series gradually decreased, and it was 13.5 and 5.5 times lower at 72 and 96 h, respectively (*p* ≤ 0.05), compared to 24 and 48 h. In the respective Kin series, the concentration of DHZR fluctuated, and its concentration was the lowest at 96 h, which was 7.25 times lower than the greatest concentration. The average concentrations of DHZR in the 24–96-h-Ctrl and 24–96-h-Kin series were approximately 6.75 and 6.75 times lower (*p* ≤ 0.05), respectively, than that at 0 h (Fig. 5D).

In the variant without meristems in the 24–96-h-Ctrl series, the concentration of DHZR fluctuated, and at 72 h, it was 4 times lower (*p* ≤ 0.05) than that at 48 h. In the respective Kin series, the concentration of DHZR gradually decreased, and at 96 h, it was 1.5 times lower (*p* ≤ 0.05) than that at 24 h. On average, the concentration of DHZR in 24–96-h-Ctrl and in 24–96-h-Kin of this variant was approximately 3 times lower (*p* ≤ 0.05) than that at 0 h (Fig. 5D’).

On average, the concentration of DHZR in the 24–96-h-Kin and 24–96-h-Ctrl series in the variant without meristems was similar (*p* > 0.05) and was approximately 3 times lower (*p* ≤ 0.05) than that in the 24–96-h-Ctrl series, and it was approximately 5 times lower (*p* ≤ 0.05) than in the 24–96-h-Ctrl and 24–96-h-Kin series in the variant with meristems (Fig. 5D,D’).

*The cZR5’MP* in the variant with meristems in the 0–96-h-Ctrl series was detected only at 0, 24 and 72 h. At 24 h, it was 2 times greater (*p* ≤ 0.05) than at 72 h. In the respective 24–96-h-Kin series, the concentration of* c*ZR5’MP fluctuated, and it was the lowest at 24 h, which was 2 times lower than the greatest value observed at 72 h in this series. On average, the concentration of *c*ZR5’MP in the 24–96-h-Ctrl and 24–96-h-Kin series was approximately 1.5 and 6 times greater (*p* ≤ 0.05), respectively, than that at 0 h (Fig. 5E).

The variant without meristems in the Ctrl series of the *c*ZR5’MP was detected only at 0, 24 and 48 h. The concentrations of *c*ZR5’MP at 24 and 48 h were similar (*p* ≤ 0.05). In the respective Kin series, the concentration of* c*ZR5’MP fluctuated, the lowest concentration was observed at 96 h, and the greatest concentration occurred at 48 h, which was 2 times greater. On average, the concentrations of *c*ZR5’MP in the 24–96-h-Ctrl and 24–96-h-Kin (approximately 1000 pmol g^−1^ DW) series were similar (p > 0.05) and 7.5 times greater (*p* ≤ 0.05), respectively, than that in the 0-h group (Fig. 5E’).

The average concentration of *c*ZR5’MP in the 24–96-h-Kin series of the variant without meristems was approximately 8 times greater (*p* ≤ 0.05) compared to its average amount in the 24–96-h-Ctrl series of this variant, and it was approximately 9.5 and 1.25 times greater (*p* ≤ 0.05) compared to 24–96-h-Ctrl and 24–96-h-Kin in the variant with meristems, respectively (Fig. 5E,E’).

### Correlation analyses

**Figure 6:**
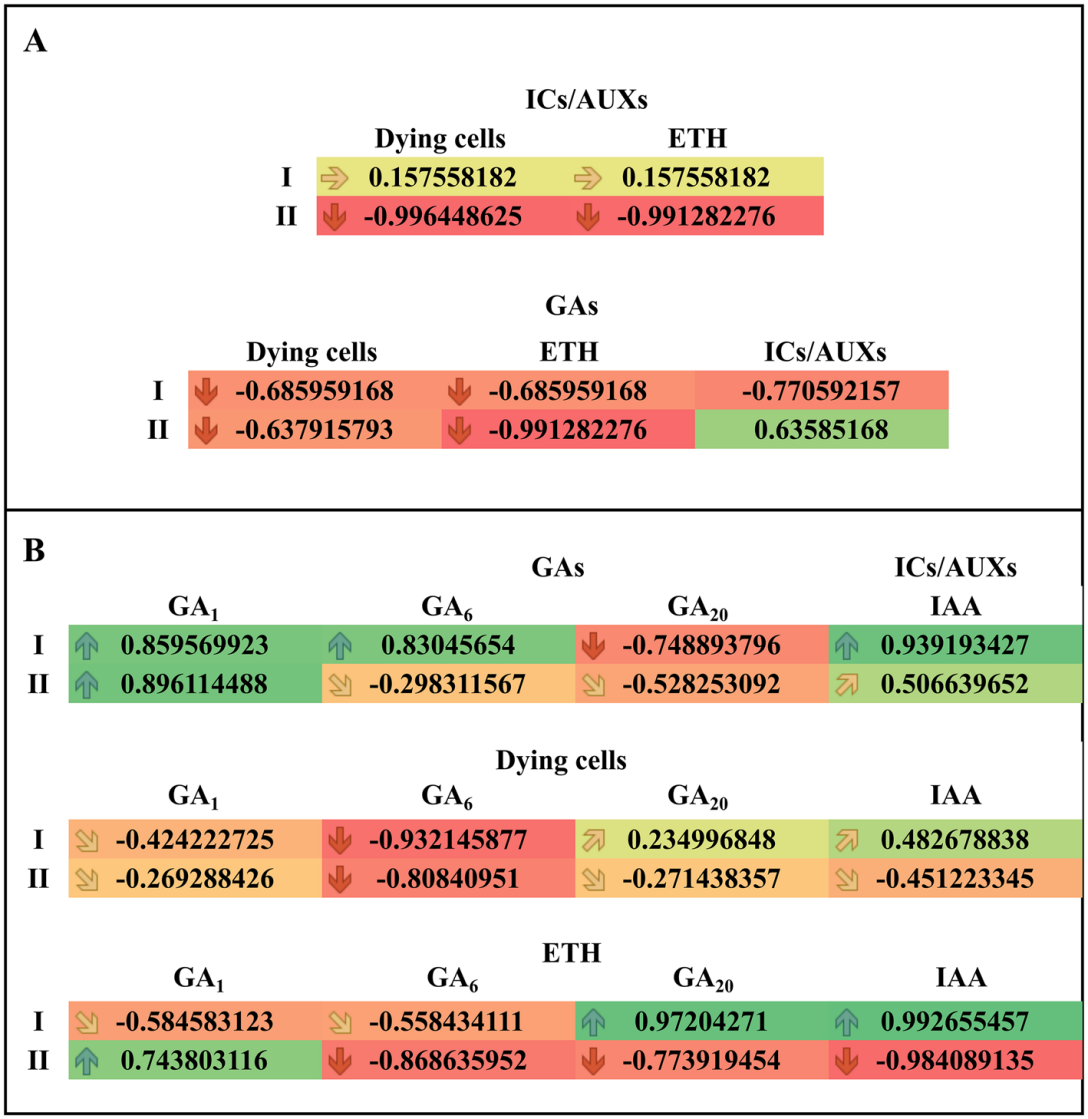
Values of Person correlations coefficients regarding the concentrations of hormones in the apical part of *V. faba* ssp. *minor* roots with meristems (I) and without meristems (II) treated with kinetin. (**A**) Correlations of the percent of dying cells (mortality) or the concentration of ethylene (ETH) with the concentration of indolic compounds/auxins (ICs/AUXs) as well as of the total concentration of gibberellins (GAs) and mortality, the concentration ETH and the total concentration of ICs/AUXs. (**B**) Correlations between the total concentration of GAs and gibberellin A_1_ (GA_1_), gibberellin A6 (GA_6_), gibberellin A_20_ (GA_20_), ICs/AUXs and indole-3-acetic acid (IAA) as well as between the concentration of gibberellin A_1_ (GA_1_), gibberellin A_6_ (GA_6_), gibberellin A_20_ (GA_20_) and IAA and mortality and the concentration of ethylene (ETH).

**Figure 7:**
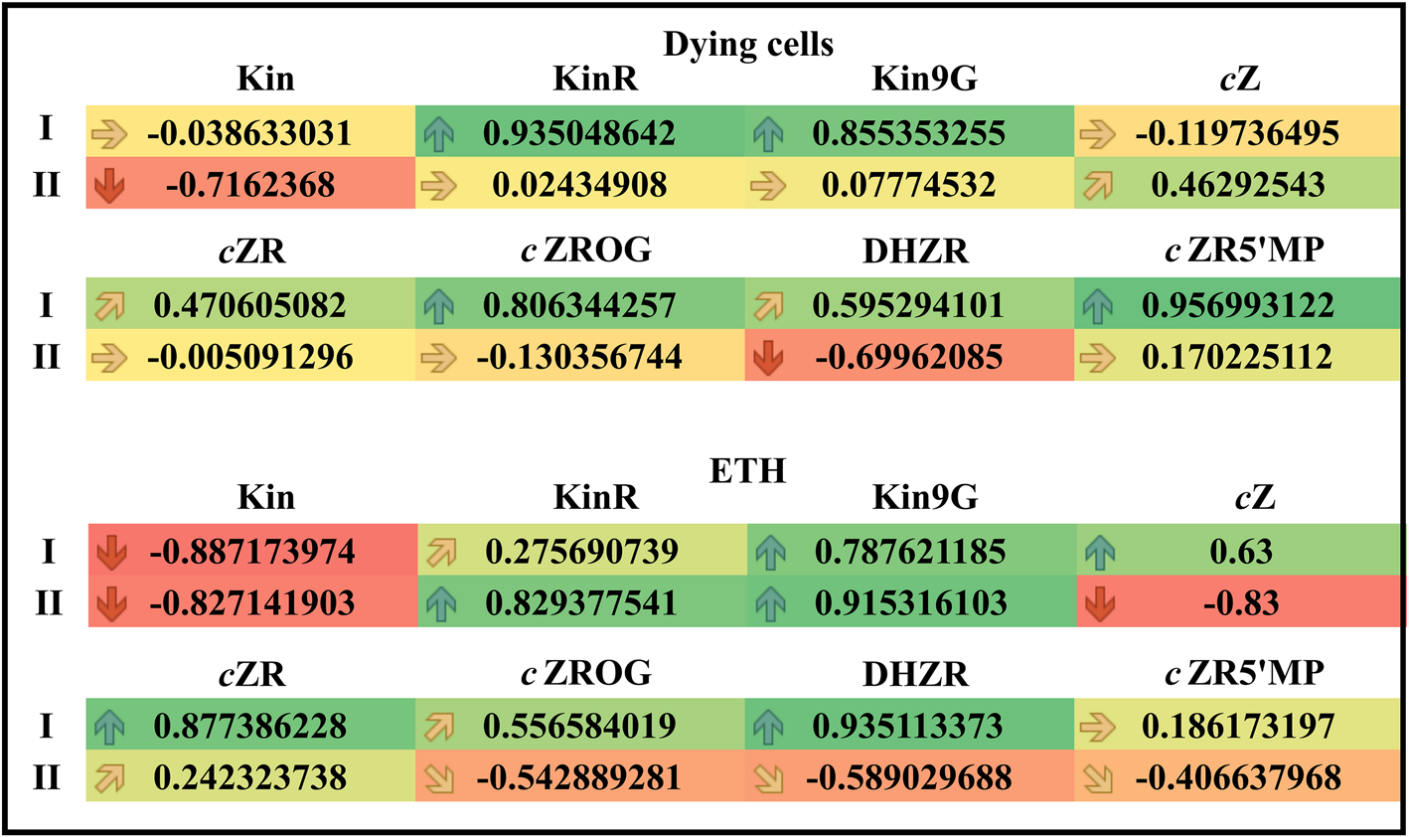
Values of Person correlations of hormone concentrations in the apical part of *V. faba* ssp. *minor* roots with (I) and without meristems (II). (**A**) Correlations between the number of dying cells (mortality) or the concentration of ethylene (ETH) and the concentration of kinetin (Kin), *cis*-zeatin (*c*Z), *cis*-zeatin riboside (cZR), kinetin riboside (KinR), kinetin-9-*O*-glucoside (Kin9G), *cis*-zeatin riboside-*O*-glucoside (cZROG), dihydrozeatin riboside (DHZR) and *cis*-zeatin-5'-monophosphate (*c*ZR5’MP).

To find the relationships between the percentage of dying cells (mortality) and plant hormones during Kin-RCD in faba bean roots, the levels of ETH, ICs/AUXs**,** GAs and CKs were subjected to analyses with Pearson’s correlation (Figs. 6 and 7).

### Correlations of mortality and the concentrations of ETH with ICs/AUXs and with total GAs as well as particular GAs and IAA with total GAs and mortality and ETH with amounts of particular GAs

There were no correlations between ICs/AUXs and mortality or ETH in the variant with meristems, while in the variant without meristems, there were strong negative correlations. There were moderate negative correlations of mortality, ETH and ICs/AUXs with the total amount of GAs in the variant with meristems and of mortality with the total amount of GAs in the variant without meristems. In this variant, the correlations between the amounts of GAs and ETH or ICs/AUXs in APR of the variant without meristems were strongly negative and moderately positive, respectively (Fig. 6A).

The total amount of GAs was not correlated only with GA_6_ in the APR of the variant without meristems, while it was moderately negatively correlated with GA_20_ in the variant with meristems and group II. There was a moderate positive correlation of ICs/AUXs with IAA in APR, the variant without meristems. In the other cases, strong positive correlations were observed.

The mortality was not correlated with GA_1_ in APR of the variant without meristems or with GA_20_ in either group. It was moderately positively correlated with IAA in the variant with meristems, negatively correlated with APR in the variant without meristems, moderately negatively correlated with GA_1_ in the variant with meristems group and strongly negatively correlated with GA_6_ in both variants with and without meristems.

The amount of ETH was moderately negatively correlated with GA_1_ and GA_6_ in the APR variant with meristems and with GA_20_ in the APR variant without meristems, while it was moderately positively correlated with GA_1_ in the latter variant. However, the correlations of the amount of ETH with GA_6_ and with IAA in APR in the variant without meristems were strongly negative, while those with GA_20_ and with IAA in the variant with meristems were strongly positive (Fig. 6B).

### Correlations of mortality and the concentration of ETH with the concentrations of particular CKs

The mortality was neither correlated with the amounts of Kin and *c*Z in the variant with meristems nor with those of KinR, Kin9G, *c*ZR*, c*ZROG and *c*ZR5’MP in the APR-II group; however, mortality was moderately positively correlated with the amounts of *c*ZR and DHZR in the variant with meristems and with* c*Z in the APR of the variant without meristems. Moderate negative correlations were observed between mortality and the concentrations of Kin and DHZR in the APR of the variant without meristems. In the cases of KinR, Kin9G, *c*ZROG and *c*ZR5’MP in the variant with meristems, strongly positive correlations were observed (Fig. 7).

The amount of ETH was not correlated with the amounts of KinR and *c*ZR5’MP in the variant with meristems group or with the amount of ***c***ZR in the APR of the variant without meristems. In the cases of the amounts of Kin9G, *c*Z and *c*ZROG in the variant with meristems, moderate positive correlations were observed, and in the cases of the amounts of *c*ZROG, DHZR and *c*ZR5’MP in the APR of the variant without meristems, moderate negative correlations were observed. Strong positive correlations were observed between the amount of ETH and *c*ZR and DHZR in the variant with meristems as well as the amounts of KinR or Kin9G in APR, but there was a strongly negative correlation between the amount of ETH and Kin in the variant with meristems.

## Discussion

Every step of plant growth and developmental programs^24^, stress responses^25^, disease syndromes and defence against pathogens depends on “crosstalk” between phytohormones. Hormonal crosstalk is also crucial for cell death^2,12,25^, which together with the cell cycle plays a central role in the process controlling plant morphogenesis. CD, in the form of PCD, i.e., developmental CD, is important for the elimination of cells during natural plant tissue formation, e.g., xylem differentiation, aerenchyma formation, development of male and female gametophytes, embryo differentiation and many other phenomena, and for the elimination of damaged cell organelles^12,26–28^. However, CD in the form of RCD^2^, i.e., externally induced CD, is a process that can be manifested by the same or similar hallmarks as those of PCD.

This paper, for the first time, presents the results of studies revealing relationships between four of nine naturally occurring groups of endogenous plant hormones^29^, i.e., ETH, ICs/AUXs, GAs and CKs (Table 2), during CD induced by exogenously applied Kin. It was shown that in the apical parts of roots with or without meristems, some of the hormones were measurable, but some of them were below detection limits (Table 2). This fact suggests that some of the hormones were not present in this specific part, or in entire plants, or that they fulfilled, or had not yet fulfilled, their roles in the first step of seedling germination (e.g., KinR at 24 h in the control series). Among the detected hormones, we found two types of changes in their amounts: (i) Kin-dependent changes, related to CD, in which the amounts of hormones differed between Kin and Ctrl series, and (ii) physiologically/developmentally dependent changes, related to the first step of seedling growth, in which the amounts of hormones in roots of Kin series were similar to those in Ctrl plants. This paper focuses only on the Kin-dependent changes in the concentrations of hormones.

Only the levels of one of the key proactive AUXs, i.e., IAA (Table 2), were shown to have relationships with Kin-dependent CD as well as with physiologically dependent processes. The amounts of IAA and the ICs/AUXs with which IAA interacts fluctuated in the Ctrl series in the roots with meristems, but were enhanced with time in the Kin series of the variant, while these parameters decreased with time in the Ctrl and Kin series of the variant without meristems. These results indicated that CKs can influence IAA concentration in apical meristems or in specific types of cells in the apical parts of roots via the induction of the signal transduction pathways of CKs and AUXs^30^. The present results suggest that a low level of IAA accompanying faba bean Kin-RCD might cooperate with GAs to control the longitudinal growth of plant cells^31^. It seems that root cortex cells in the Kin series were shorter compared to the Ctrl series due to the relatively low level of GAs^8^. However, it is also known that GAs and CKs exert antagonistic effects on root elongation^32^.

The results of Doniak et al.^11^ showed that altered cell dimensions are important morphological features of Kin-RCD in faba bean; they found that the W (width)/L (length) ratio of cortex cells in 72-h-Kin group was 5.0, but 2.5 for the 72-h-Ctrl group. A change in cell dimensions is characteristic of cell differentiation, for instance, of antheridial mother cells undergoing asymmetrical division to form antheridial initial cells; here, the W/L ratio reached a value of approximately 1.6^33^. The phenomenon related to maintenance of the specific cell W/L ratio was also observed during the differentiation process leading to xylem formation, in which PCD plays a crucial role^26^.

Indeed, our results showed that in the Kin series of the variant with meristems, GA_20_ levels increased over consecutive days, while in the 24-h-Ctrl group, the level of GA_20_ was higher than that in the 0-h-Ctrl group, but then decreased over time. On the other hand, in the 24–96-h-Ctrl series of the variant with meristems, the concentrations of GA_20_ were greater than those at 0 h, but in the Kin series of the variant without meristems, its amounts were similar throughout the experiments, showing that Kin might reduce the extent to which the concentration of GA_20_ is elevated during the following days compared to the Ctrl series. Thus, decreasing levels of bioactive forms of GAs, i.e., GA_6_ and GA_1_, acting with Kin directly or indirectly through the other CKs, may block the conversion of GA_20_ into the active forms of GAs because in the Kin series of the variants both with and without meristems, the concentrations of GA_6_ and GA_1_ were similar to those in the Ctrl series with and without meristems. The meaning of these results was enhanced by the lack of a significant correlation of these GAs with mortality.

The reduction in the amount of bioactive GAs over time may be the result of an increase in the amount of ETH, which is important for the regulation of cell expansion^11^. This is how CKs counteract the physiological activities of GAs. Thus, the results allow us to suggest that the greater amount of GA_20_ at the first stage of faba bean Kin-RCD might be one of the important hallmarks of the process explaining the process induction, as is the case for PCD during endosperm development^34^ and at the first steps of seed germination^34,35^. Moreover, higher levels of GAs in the Kin series of the variant with meristems compared to that without meristems indicated that the root meristem was a site of biosynthesis and/or metabolism^36^ of GAs.

In the variant of the Kin-treated seedlings with meristems, the amount of Kin reached approximately 20,000 pmol g^−1^ DW, while in the Kin series of the variant without meristems, 30,000 pmol g^−1^ DW was achieved. In the variants with and without meristems, the concentrations of Kin declined over time, with the lowest values being observed in 96-h-Kin group in the variant with meristems and in 72-h-Kin in the variant without meristems. These results indicated that Kin was converted into other forms of CK, e.g., KR and/or Kin9G^37^. Indeed, measurable amounts of KinR and Kin9G in the Kin series of the variant with meristems appeared after 24 h of treatment, and their levels reached approximately 4000 pmol g^−1^ DW; these levels did not change throughout the experiment. Moreover, the amount of KR in the Kin series of the variant without meristems was several times greater than that in the Kin series of the variant with meristems. This result suggests that KR plays an important role in the induction of faba bean Kin-RCD in the root cortex because some of the cortex cells die after Kin application. This suggestion could support the hypothesis that meristems play an important role in the metabolism of Kin and/or its metabolites^38,39^.

This was validated by the positive Pearson correlations of the analysed CKs, except Kin, with mortality observed in both variants, and by the lack and negative correlations of the amount of Kin with mortality in the Kin series with and without meristems, respectively. This hypothesis confirms studies on soybean callus culture, in which Kin was converted to KinR within 48 h^37^.

It was established that the Kin-RCD in the faba bean reached its culmination at 72 h of Kin group^8^, when the most significant number of dying cells (approximately 45%) was observed, while in 24-h, 48-h and 96-h Kin groups, the corresponding numbers were 0%, 35–40% and 30–35%, respectively. This may be related to the possible role of Kin in the inhibition of cell division during the G1 phase of the cell cycle in root meristems and in the induction of CD in the cortex^8^. The cell cycle inhibitory effect of KR might be evoked through *c*ZR, which, when applied at the early G1 phase, can arrest the cell cycle^40^. Although there are many studies in the scientific literature indicating that *cis*-zeatin derivatives have no biological activity, Kudo and coworkers (2012) reported that both *cZ* and *c*ZR inhibited seminal root elongation and upregulated cytokinin-inducible genes. The activities of these CKs were comparable to those of *t*Z. The authors showed that supplied *c*ZR was converted mainly into other *cis* derivatives, i.e., *c*ZOG, which, similar to cZROG, is a storage form of *cis* metabolites of CKs^41^.

The present results suggested that *c*Z was synthesized in meristems because of observed levels which were several times greater in the Kin series of the variant with meristems than in that without meristems. In the Kin series of the variant with meristems, *c*Z was found to be converted to *c*ZR, *cZ*ROG and DHZR because the amounts of *cZ* and *cZ*ROG in the Kin series of the variant with meristems and of *cZ*ROG and DHZR in the Ctrl series of the variant without meristems were the greatest in 3-d-old seedlings in 0-h-Ctrl group, and over the following days in the Ctrl series of the variants with and without meristems, their amounts decreased with time. The amount of *c*Z increased with time in the Kin series of the variant without meristems, reaching a maximum at 72 h; this was positively correlated with mortality because the process reached culmination at this time, reflected by the greatest mortality.

The results also indicated that the levels of *c*ZR and *cZ*ROG were greater in the Kin series of the variant with meristems in comparison with the Kin series of the variant without meristems. The concentration of *c*ZR in the Kin series of the variant with meristems fluctuated, and although its greatest level was observed in 72-h-Kin, the average amount was lower than in the 0–96-h-Ctrl series of the variant with meristems; however, in the variant without meristems, the average concentration of *c*ZR was greater than that in the 0–96-h-Ctrl series of the variant without meristems. The average amounts of *cZ*ROG in the Kin series of the variants with and without meristems were greater than those at 0–96 h in the Ctrl series of the variants with and without meristems.

These facts suggested that *c*Z*, c*ZR and *cZ*ROG, but not DHZR, might participate in Kin-RCD in the faba bean program. It should be noted that root cortex cells do not divide, and *c*Z is a factor that has been shown to be necessary for the G2/M transition in tobacco cells^40^. Thus, CD induction resulted from the fact that the cell cycle could not be activated. Therefore, mitotic catastrophe might have occurred during the RCD of faba bean, as was proven during RCD in BY-2^7^.

The scientific literature suggests that *cis* derivatives of zeatin in rice can be transformed to *trans* derivatives and vice versa^41^. However, the present results, which indicated that *cis* derivatives were present in meagre concentrations or were undetectable (Table 2), might indicate that they were not transformed to *trans* derivatives or that conversion occurred, and *trans* derivatives were quickly metabolized in some other way. Thus, *trans* metabolites of zeatin may serve only physiologically dependent roles related to the next steps of growth of faba bean seedlings, which suggests that the *cis-*dependent pathway downregulates *trans-* and iP-dependent pathways. These findings are in agreement with the earlier supposition that Kin-RCD in faba bean is a very specific process^5,6,42^ and that *cis* forms of zeatin derivatives are involved in it.

Cytokinin nucleotides, e.g., BAP monophosphate, were shown to be more active than free CK bases in controlling CD^21,43^. We previously hypothesized that during Kin-RCD in faba bean, Kin was converted into Kin monophosphates (Kin MP)^8^ via phosphoribosyltransferase^21^ and that MPs of CKs might induce CD^8,21^. These results indicated the presence of one of the MPs, i.e., cZR5’MP, in similar average quantities in the Kin series in the variants with and without meristems (Fig. 5E,E’); however, its levels were greatest in the 48-h-Ctrl and 72-h-Ctrl groups in the variants with and without meristems, respectively, and its concentration gradually decreased with time. The concentration of this CK was strongly positively correlated with mortality in the Kin series of the variant with meristems and slightly positively correlated in the Kin series of the variant without meristems. These facts strongly suggested that *c*ZR5’MP is the direct factor triggering Kin-RCD in faba bean: in the Kin series both with and without meristems, the concentration of *c*ZR5’MP exhibited a similar profile, indicating that the signal was generated in meristems, and it was spread through the whole apical part of roots, leading to death only of selected cells.

**Figure 8:**
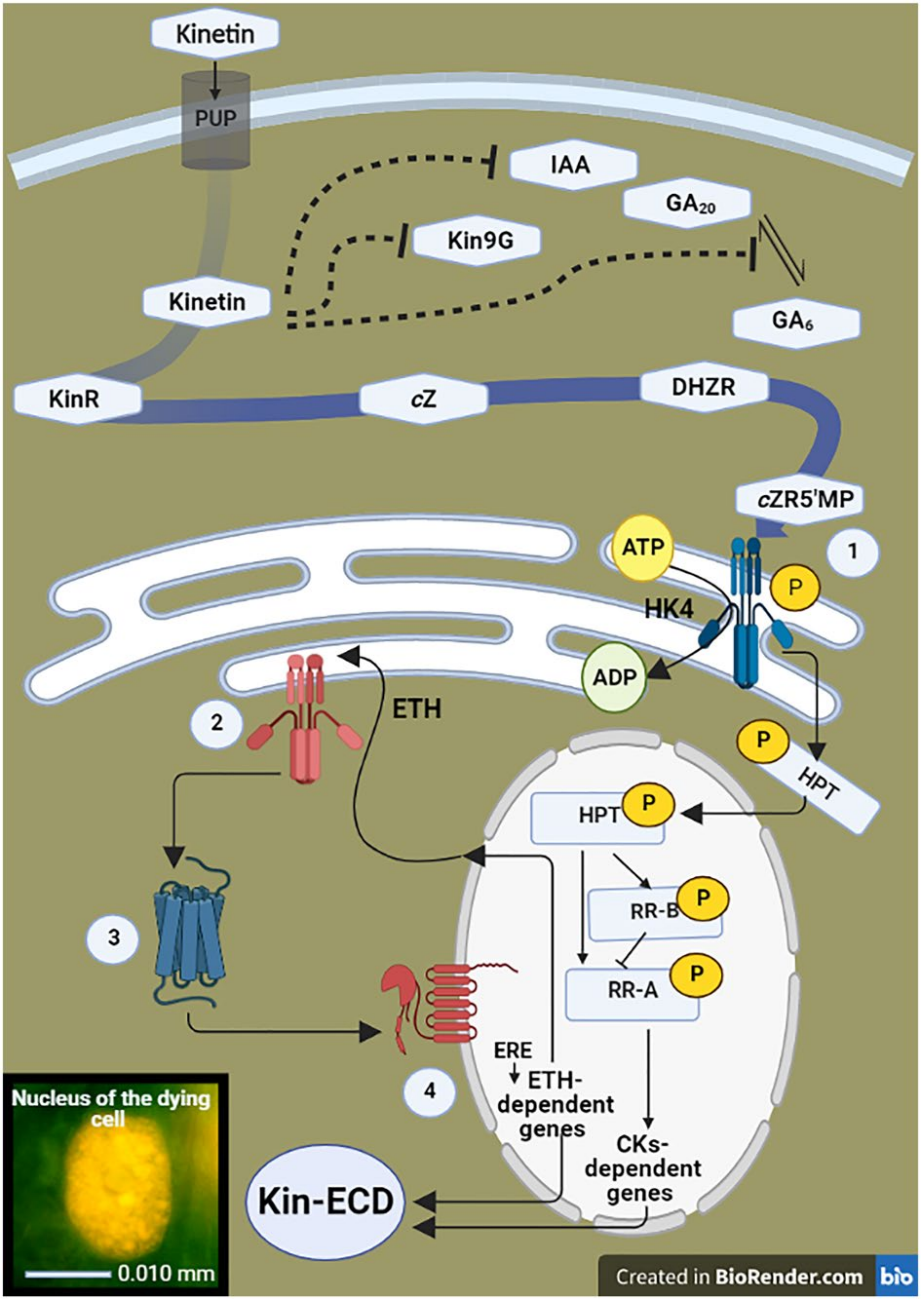
Scheme of crosstalk between kinetin (Kin), *cis*-zeatin (*c*Z), *cis*-zeatin riboside (*c*ZR), *cis*-zeatin riboside-*O*-glucoside (*c*ZROG), dihydrozeatin riboside (DHZR), *cis*-zeatin-5'-monophosphate (*c*ZR5’MP), ethylene (ETH), indole-3-acetic acid (IAA) and gibberellin A_20_ (GA_20_), gibberellin A_6_ (GA_6_) and gibberellin A_1_ (GA_1_) during the control of induction and course of cell death of in the root cortex of *V. faba* ssp. *minor* seedlings (Kin-RCD), based on the suggestions presented by Kunikowska et al.^7^, Doniak et al.^8^ and Kaźmierczak et al.^20^. Kinetin transported to the cell through purine transporter (PUP) is converted to KinR, then to cZ, DHZR and *c*ZR5’MP, which activates histidine kinase (HK) receptors (i.e., HK4) and the ethylene (ETH)-dependent signalling pathway using histidine-containing phosphotransmitter (HPT) leading to aerenchyma cavities formation. Simultaneously, HPT may stimulate a cytokinin response regulator (RR-B) and by activating ETH-dependent genes through ethylene response elements (EREs) may increase ETH synthesis, enhancing the PCD effect. Moreover, Kin, directly or indirectly, counteracts the effects of IAA, Kin9G and GA_6_. (1) Cytokinin histidine kinase 4 (HK4) receptors; (2) ETH histidine kinase receptors (ETR/ERS/EIN4); (3) receptor activating constitutive triple response 1 (CTR1), a RAF-like serine/threonine (Ser/Tr) kinase; (4) ethylene insensitive (EIN2) nuclear transmembrane protein.

The results of the paper allowed us to propose a probable scheme of Kin-induced CD regulation by ETH, ICs/AUXs, IAA, GAs and CKs. The scheme by which Kin induces Kin-RCD in faba bean may be as follows: 48 h after application of Kin, Kin is metabolized to Kin9G and KR in the culture solution of growing seedlings, and the latter triggers an increase in the concentrations of *c*Z and *c*ZR in the Kin series of the variants with and without meristems, respectively. Next, these compounds stimulate an increase in DHZR and *c*ZR5’MP concentrations. The latter can activate the HK4 cytokinin receptor, which is suggested in the scientific literature to be the death receptor^21,37^. These findings, once again, strongly indicate that the *cis*-zeatin-dependent pathway is responsible for the control of the Kin-dependent CD process (Fig. 8).

DHZR may take part in the regulation of cell growth and cell wall degradation^44^, while *c*ZR5’MP could activate downstream serine proteases because their greatest activities were observed during the signalling phase of Kin-RCD in the faba bean. These proteases could stimulate the activities of catabolic cysteine proteases^6^ and then nucleases^38^, fluctuations in the activities of which were observed leading to induction of protoplast digestion. This is possible because inhibitors of adenosine kinase that block the formation of monophosphates from N^6^-substituted derivatives of adenosine completely prevent cells from undergoing apoptosis^21^. Moreover, Kin, acting via the calcium ion-dependent^10^ pathway that stimulates ETH synthesis^45^, could lead to an increase in the concentration of ETH, and ETH cooperation with Kin might limit longitudinal growth and stimulate both thickening of cells and cell wall degradation during the RCD pathway in faba bean.

However, the question remains: what is the key factor of the process? To clarify this issue, it is important to understand the role of CKs in controlling DNA endoreplication. When cells in roots reach the transition zone, they stop dividing and enter the endocycle via activation of two transcription factors, the type-B Arabidopsis response regulators 1 and 12 (ARR1 and ARR12) and induction of indole-3-acetic acid inducible nr 3, short hypocotyl (SHY2/IAA) member of the Aux/IAA family repressor of AUXs signalling^46^.

This paper presents the results indicating low levels of IAA and high levels of CKs, which reflect the arrest of meristem dividing cells in the G1 phase of the cell cycle^8^. Takahashi et al. reported^46^ that this phenomenon was related to the degradation of anaphase-promoting complex/cyclosome (APC/C) via E3 ubiquitin ligase. This ligase is activated by cell cycle switch52 A1 (CCS52A1), which is directly upregulated by CK-activated ARR2. The fact that CD and micronuclei and/or apoptotic-like bodies are observed in the cortex cells of faba bean roots after Kin treatment^6^, where there is no cell division, suggests that these cells remove the excess DNA.

In animals, the formation of apoptotic bodies is the key process activating mitotic catastrophe, during which cells may die^2,47^. This may be important to prepare nuclei with appropriate DNA content for the division of endoreduplicated cells by the deduplication process, and this is in agreement with the results published by Kunikowska et al.^8^, who observed cell cycle arrest and a greater number of endoreplicated cortex cells in Kin-treated roots. Similar phenomena were proven in CD induced by Kin in BY-2 cells in suspension culture during the studies carried out by Kaźmierczak and coworkers in 2023^7^. This process was also manifested by microtubular and actin breakdown^7^.

In conclusion, the results-based analyses of the concentrations of ETH, ICs/AUXs, GAs and CKs allowed us to conclude that crosstalk between them is responsible for the induction and progression of Kin-RCD in the cortex of roots of *V. faba* ssp. *minor* seedlings. Among more than 40 individual compounds, only ETH, IAA, GA_20_, GA_6_, GA_1_, KinR, Kin9G, *c*Z, *c*ZR, *c*ZROG, DHZR and *c*ZR5’MP as well as total indolic compounds, to which IAA belongs, and total and individual GA compounds responded to Kin application by changing their concentrations during four days of treatment.

These results strongly indicated that the *cis*-zeatin-dependent pathway, but not the *trans*-zeatin-dependent pathway, is responsible for controlling the induction and progression of Kin-RCD in faba bean because *c*ZR5’MP is a candidate ligand that is able to activate one of the CKs strongly suggested as the histidine CD receptor, i.e., HK4^21,37^, while KinR might be a key factor in initiating the cascade of events related to the process.

Moreover, the fact that the primary function of Kin is stimulation of cell division^40^, as shown in this paper, as well as in previous works related to roots of faba bean^5,6,42^ and BY-2 suspension^7^, proved that Kin in both systems induced CD, which is accompanied by cycle arrest and induction of endoreplication. Taken together, this allowed us to conclude that the main background of the process may be mitotic catastrophe.

## Material and methods

### Plant material, treatment, morphological measurements and sample preparation

*Vicia faba* ssp. *minor* cv. ‘Bobas’ seeds (40) were germinated in a dark incubator in Petri dishes (Φ300 mm and H 30 mm) on two layers of blotting paper moistened with distilled water (1.5 mL per seed) for 3 days. Then, fifteen seedlings (Fig. 1A), with root lengths of approximately 20 ± 5 mm, were transferred into Erlenmeyer flasks (1 L, Φ120 mm and H 210 mm) with two layers of blotting paper with 25 mL of either water or an aqueous solution of 46 μM Kin and cultivated at 23 ± 1 °C and 92 ± 2% relative humidity for 96 h. Laboratory equipment was sterilized using Varioclav (https://animalab.pl).

The ETH concentrations were measured in the surrounding air of the seedlings, from which one-third of the APR (Fig. 1) was used to analyse the mortality of cortex cells and for the qualitative and quantitative analyses of ICs/AUXs, GAs and CKs, which were performed in apical parts of roots with or without meristems of the control seedlings cultivated for 0, 24, 48, 72 and 96 h and treated with Kin for 24, 48, 72 and 96 h. To obtain the fragments with meristems (Fig. 1), one-third of the APR was cut off, while to obtain the fragments without meristems, one-third of the APR was cut off and then shortened into 3-mm parts with meristems (Fig. 1), which were cut off.

### ETH concentration measurement

For measurements, Erlenmeyer flasks were tightly capped with pipette tips inside. After 30 min of cultivation in the dark, the SCS56 (the ETH analyser; Storage Control System, United Kingdom) was connected via a flexible tube, and measurements were conducted for 30 s. The results from the 20th to 30th s were taken to calculate the ETH concentration in ppm per seedling.

### Cell mortality identification and estimation

To estimate the percentages of living, dying and dead cells, the fluorescent nuclear test was applied^8,20^. One-third of apical fragments of Ctrl and Kin-treatedroots were washed twice with 10 mmol L^−1^ Na phosphate buffer at pH 7.4 (PHB) and stained with a mixture of 100 µg mL^−1^ acridine orange (AO) and 100 µg mL^−1^ ethidium bromide (EB) in PHB for 5 min. Then, the fragments were washed twice with PHB, fixed with 2.5% (v/v) glutardialdehyde (Merck) in PHB for 15 min, cut into very thin sections along their long axes, washed three times with PHB, analysed using a fluorescence microscope and photographed.

The colour of chromatin fluorescence changes from green for AO (migrating into the nuclei in each case) to red for EB (nonpermeable through the intact cellular membrane). Thus, the amount of EB increases in nuclei with the loss of cell membrane integrity, leading to greater red fluorescence of nuclei^8,20,21^. For this paper, expanded classification of colour smoothly passing from green to red was performed.

We observed (1) green and green‒yellow nuclear chromatin in living cells, (2) yellow, yellow‒orange, bright-orange and orange chromatin in dying cells and (3) dark-orange and red chromatin in dead cells (Supplementary Fig. S2). The resultant fluorescence intensity (R.F.I.) values of these groups ranged from 10 to 35, above 35 to 45 and above 45 to 55 a.u., respectively. According to these parameters, R.F.I. of 35–40 a.u. indicates the first stage of cell death (CD-I), while 40–45 a.u. indicates the second stage (CD-II). Additionally, during CD-I, condensation and fragmentation of nuclei^9^ started, and during CD-II, these processes continued during CD-II.

Measuring the resultant fluorescence intensity (R.F.) of nuclei using Scion Image and attributing its values according to a previously^9,20,21^ prepared scale allowed us to calculate the numbers of living, dying and dead cells, as well as dying cells in CD-I and CD-II.

### Quantitative analysis GAs (total and individual)

To determine the concentration of total GAs, the samples were prepared by freezing on dry ice and homogenization of fresh apical parts of roots (approximately 100–200 mg per sample) in an Epi-like tube using a plastic pestle with 0.05 M Tris–HCl buffer (pH 7.4). After extraction, re-extraction and centrifugation at 5000*g,* isolation was conducted according to Kaźmierczak (1999) using methanol and acidic ethyl acetate^48^.

Then, 50-μL samples dissolved in methanol were supplemented with 1.5 mL of freshly prepared Folin-Wu reagent, which consisted of phosphomolybdic acid (7 g), 40 mL NaOH (10%), sodium tungstate (1 g) and 25 mL of phosphoric acid (89%) in 100 mL. The mixtures were incubated for one hour at 100 °C. After cooling, the absorbance at 780 nm was measured. For the final calculations of the GA concentration, a calibration curve with standard gibberellic acid (GA_3_; Merck) ranging from 1 to 100 µg was prepared^49^.

To determine individual gibberellins, sample preparation was performed according to the modified method of Urbanová et al.^50^. Freeze-dried plant tissue samples (5–10 mg) were ground to a fine consistency using 3-mm zirconium oxide beads (Retsch GmbH & Co. KG, Haan, Germany) and extracted with an MM 301 vibration mill at a frequency of 30 Hz for 3 min (Retsch GmbH & Co. KG, Haan, Germany) with 1 mL of ice-cold 80% acetonitrile containing 5% formic acid as an extraction solution. The samples were then extracted overnight at 4 °C using a Stuart SB3 benchtop laboratory rotator (Bibby Scientific Ltd., Staffordshire, UK) after adding 17 internal GA standards ([^2^H_2_]GA_1_, [^2^H_2_]GA_3_, [^2^H_2_]GA_4_, [^2^H_2_]GA_5_, [^2^H_2_]GA_6_, [^2^H_2_]GA_7_, [^2^H_2_]GA_8_, [^2^H_2_]GA_9_, [^2^H_2_]GA_15_, [^2^H_2_]GA_19_, [^2^H_2_]GA_20_, [^2^H_2_]GA_24_, [^2^H_2_]GA_29_, [^2^H_2_]GA_34_, [^2^H_2_]GA_44_, [^2^H_2_]GA_51_ and [^2^H_2_]GA_53_; purchased from OlChemIm, the Czech Republic). The homogenates were centrifuged at 36,670*g* at 4 °C for 10 min, and the supernatants were further purified using reversed-phase and mixed mode SPE cartridges (Waters, Milford, MA, USA) and analysed by ultrahigh-performance liquid chromatography-tandem mass spectrometry (UHPLC‒MS/MS; Micromass, Manchester, U.K.). The individual GAs were detected and quantified using the multiple-reaction monitoring mode according to the transition of the ion [M–H]^−^ to the appropriate respective ion product by MassLynx 4.1 software (Waters, Milford, MA, USA), and the standard isotope dilution method^51^ was used.

### Quantitative analysis of ICs/AUXs, AUXs, IAA and CKs

The measurements of the total concentration of ICs/AUXs were conducted using the extracts prepared for estimation of total GAs, and the method with Salkowski reagent was adopted. The prepared 500-μL samples dissolved in methanol were supplemented with 1.0 mL of Salkowski reagent, which consisted of FeCl_3_ (12 g) in 1 L of H_2_SO_4_ (7.9 M). The mixture was incubated for 30 min at room temperature, and the absorbance at 780 nm was measured. For the final calculations of the concentration of ICs/AUXs, a calibration curve with standard IAA (Merck) from 1 to 100 µg was used^52^.

To analyse the levels of individual AUXs and CKs, lyophilized plant tissues were used. Triplicates of each sample (10–15 mg) were extracted in 1 mL of modified Bieleski buffer (60% MeOH, 10% HCOOH and 30% H_2_O) with stable isotope-labelled ^3^H-CKs (0.25 pmol per sample of CK bases, ribosides, 9-glucosides and 7-glucosides and 0.5 pmol per sample of CK nucleotides) and AUXs (5 pmol per sample) internal standards to control the purification step and to validate the accuracy of determination^53^. The samples were purified using an adjusted version of the purification protocol^54^ with MCX cartridges (30 mg mL^−1^), which were used to collect the AUXs fraction. CK and AUX fractions were evaporated with a Speed Vac concentrator and dissolved in 30 µL of 10% (v/v) MeOH. The samples were analysed on an ultra-performance liquid chromatograph (Acquity UHPLC® I-class System; Waters, Milford, MA, USA) coupled to a triple quadrupole mass spectrometer (MS/MS; Xevo™ TQ-S, Waters, Manchester, UK) equipped with an electrospray interface (ESI) and Kinetex 1.7u C18 100A, 50 × 2.1 mm column (Phenomenex) using analytical separation for CKs^55^ and AUXs^55^. Quantification was carried out by multiple reaction monitoring of [M+H]^+^ and the respective product ion. Optimal conditions, including dwell time, cone voltage, and collision energy in the collision cell, corresponding to the exact diagnostic transition, were determined for each CK and AUX for selective MRM experiments^55,56^. Quantification was performed by MassLynx4.1 software (Waters, Milford, MA, USA) using a standard isotope dilution method.

### Statistical analysis and software

Three biological replicates, at least in triplicate or duplicate and more random samples, were analysed. The samples were prepared from at least six plants. The results of measurements were statistically verified by the Mann–Whitney U test and/or Student’s t test using MS Excel software (licenced) by independent step-by-step analyses of each of the results. Significant differences between results were observed at *p* ≤ 0.05.

MS Excel was also used for chart preparation and Pearson’s coefficient calculation and presentation. Values of Pearson’s coefficient, *r*_xy_, between 0.0 and 0.4 (from orange to yellow-coloured cells with horizontal and upward slanting arrows) or 0.0 and − 0.4 (from orange to light salmon-coloured cells with horizontal and downward slanting arrows), 0.41 and 0.8 (from yellow to yellow-green-coloured cells with upward slanting and vertical arrows) or − 0.8 and − 0.41 (from light salmon to dark salmon-coloured cells with downward slanting and vertical arrows), as well as values greater than 0.81 (green-coloured cells with vertical arrows) or below − 0.81 (red-coloured cells with vertical arrows) indicate no, moderate, and strong correlations, respectively. Values of r_xy_ greater than 0.41 were considered statistically significant at *p* ≤ 0.05.

The viability of cells was assessed under an epifluorescence microscope (Optiphot-2, Nikon, Japan) equipped with a B2A filter, DDX 1200 camera and ACT-1 software (Precoptic, Poland; https://precoptic.pl). To estimate the numbers of living, dying and dead cells, Scion Image (https://scion-corporation.software.informer.com) software for Win 10 was used.

Quantification of CKs, AUXs and GAs was performed using MassLynx 4.1 software (Waters, Milford, MA, USA).

The manuscript was written using MS Word, while Picosmos Tools (open source), CorelDRAW GRAPHICS SITE X7 EDU LIC (licenced) and/or INKSCAPE (open source; https://inkscape.org) were used to prepare figures and image planes in PNG extensions. Images of seedlings were taken using a Canon 100 (Japan) private camera in the JPG extension.

Moreover, BIORENDER software was used to prepare Fig. 8. Preparations of citations and the list of references were conducted with the help of the Open Mendeley Website.

### Confirmation

Authors confirmed that permission of usage of seeds of *V. faba* spp. *minor* var. ‘Bobas’ for scientific application in the Department of Cytophysiology, Pomorska 141/143, 90-236 Łódź, Poland have been bought in Polish commercial company, i.e., Danko, Hodowla Nasion Sp. z o.o. (http://www.danko.pl).

### Declaration

The authors confirm that all experiments with seedlings of nongenetically modified *Vicia faba* ssp. *minor* were performed in accordance with relevant guidelines and regulations, using operation instructions for the laboratory equipment and measuring instruments. All applied study methods were performed in accordance with the relevant guidelines and regulations using protection equipment against hazards. All images of the manuscript have been taken by the authors and all figures of the paper are the effects of the authors collaborations.

### Statement of compliance

Experiments on plants in the present study were performed with international, national and/or institutional guidelines. It means that the number of seeds used to germination was depended on seed germination rates and it was directly related with the numbers of seedlings planned to use during experiments.

## Supplementary Information

Supplementary Figures.

## Data Availability

The datasets generated and analyzed during the current study are available from the corresponding author on reasonable request.
