# Supplementary material for: Hormonal crosstalk controls cell death induced by kinetin in roots of Vicia faba ssp. minor seedlings
Source: Sci Rep. 2023 Jul 19;13:11661. doi: 10.1038/s41598-023-38641-5 (PMC10356926; doi:10.1038/s41598-023-38641-5)
Supplement: Supplementary file 1 — Supplementary Figures. [file 41598_2023_38641_MOESM1_ESM.docx]

**Supplementary Figures**


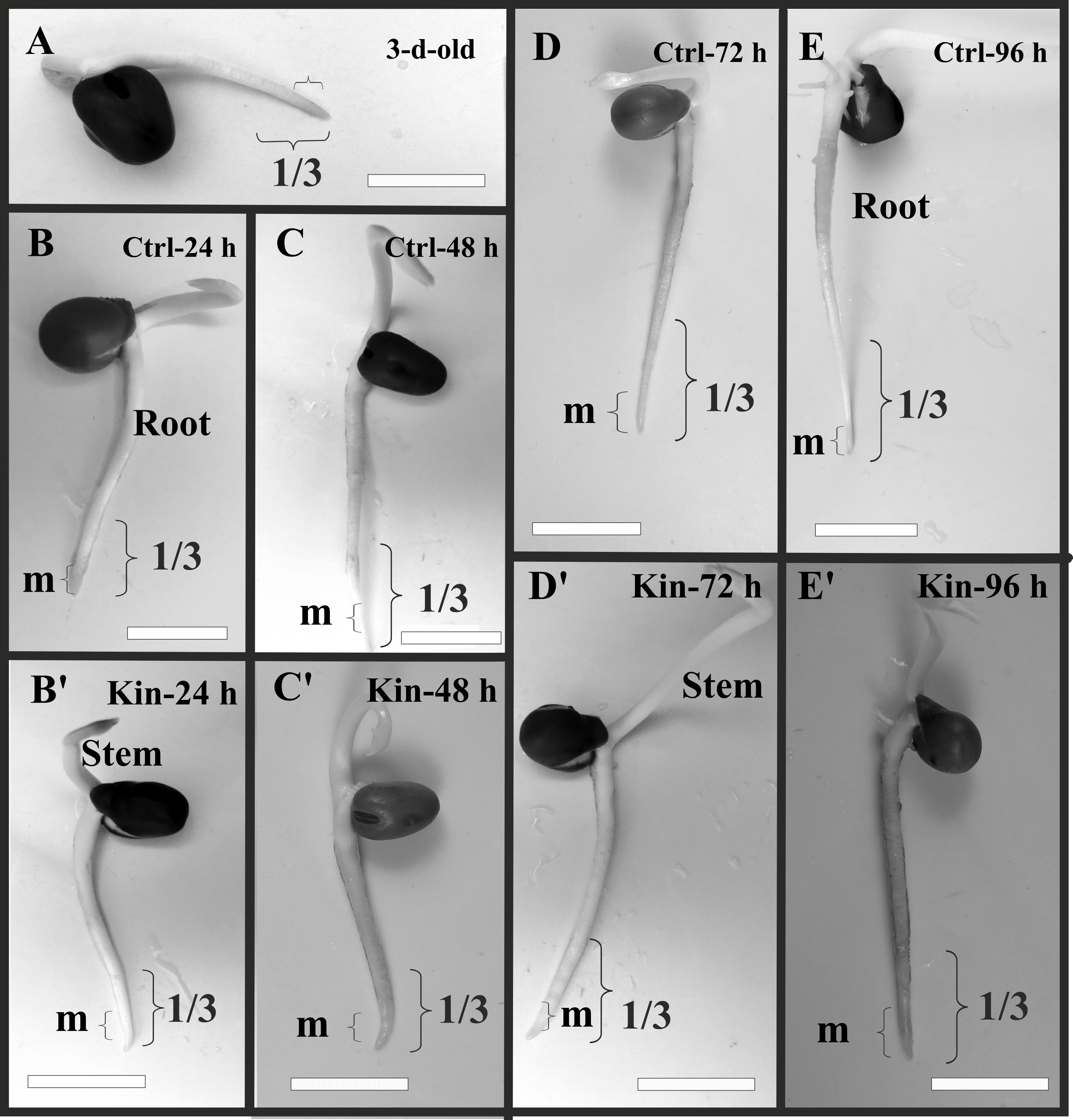


**Figure S1.** Representative 3-day-old (0 h; A) *V. faba* ssp. *mino*r seedlings cultured in control (Ctrl) conditions (B–E) and treated with Kin (B’–E’) for 24–96 h. m, meristem; 1/3, one third, of apical parts of roots. Scale bars = 10 mm.

| 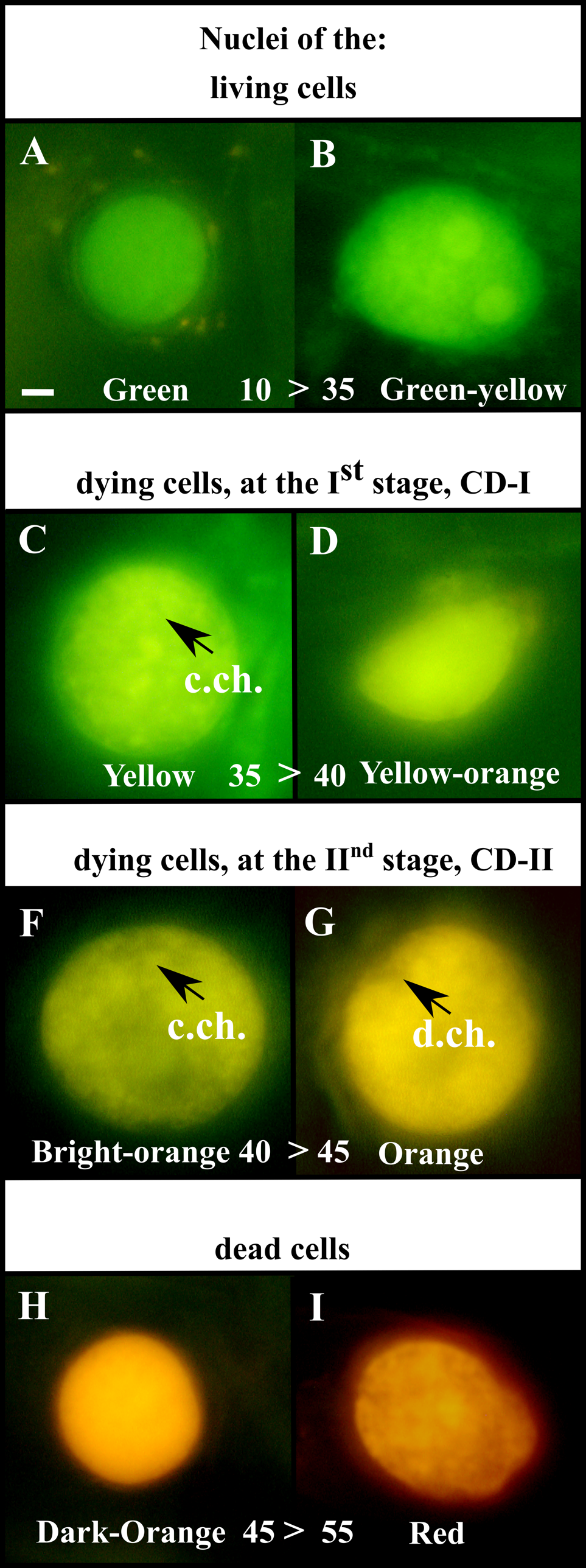 |
| --- |
| **Figure S2.** Images presented extended, compared to the Kaźmierczak et al. 2021 paper, classification green (A) and green-yellow (B) nuclei of the living cells, yellow (C) and yellow-orange (**D**) as well as light-orange (F) and orange (G) of the dying cells respectively during the first stage (CD-I) and the second stage (CD-II) of the of the process, and dark-orange (H) and red (I) nuclei of the dead cells. The numbers in the images showed the values of respective fluorescence intensity (R.F.I). Scale bars in A = 2 µm apply to all images. Arrows indicate condensing (c.ch.) and degrading (d.ch.) chromatin. |
